# METABOLIC: high-throughput profiling of microbial genomes for functional traits, metabolism, biogeochemistry, and community-scale functional networks

**DOI:** 10.1186/s40168-021-01213-8

**Published:** 2022-02-16

**Authors:** Zhichao Zhou, Patricia Q. Tran, Adam M. Breister, Yang Liu, Kristopher Kieft, Elise S. Cowley, Ulas Karaoz, Karthik Anantharaman

**Affiliations:** 1 Department of Bacteriology, University of Wisconsin-Madison, Madison, WI 53706 USA; 2 Department of Integrative Biology, University of Wisconsin-Madison, Madison, WI 53706 USA; 3 Archaeal Biology Center, Institute for Advanced Study, Shenzhen University, Shenzhen, Guangdong 518060 China; 4 Microbiology Doctoral Training Program, University of Wisconsin-Madison, Madison, WI 53706 USA; 5 Earth and Environmental Sciences, Lawrence Berkeley National Laboratory, Berkeley, CA 94720 USA

**Keywords:** Functional traits, Metagenome-assembled genomes, Microbiome, Biogeochemistry, Metabolic potential, Microbial functional networks

## Abstract

**Background:**

Advances in microbiome science are being driven in large part due to our ability to study and infer microbial ecology from genomes reconstructed from mixed microbial communities using metagenomics and single-cell genomics. Such omics-based techniques allow us to read genomic blueprints of microorganisms, decipher their functional capacities and activities, and reconstruct their roles in biogeochemical processes. Currently available tools for analyses of genomic data can annotate and depict metabolic functions to some extent; however, no standardized approaches are currently available for the comprehensive characterization of metabolic predictions, metabolite exchanges, microbial interactions, and microbial contributions to biogeochemical cycling.

**Results:**

We present METABOLIC (METabolic And BiogeOchemistry anaLyses In miCrobes), a scalable software to advance microbial ecology and biogeochemistry studies using genomes at the resolution of individual organisms and/or microbial communities. The genome-scale workflow includes annotation of microbial genomes, motif validation of biochemically validated conserved protein residues, metabolic pathway analyses, and calculation of contributions to individual biogeochemical transformations and cycles. The community-scale workflow supplements genome-scale analyses with determination of genome abundance in the microbiome, potential microbial metabolic handoffs and metabolite exchange, reconstruction of functional networks, and determination of microbial contributions to biogeochemical cycles. METABOLIC can take input genomes from isolates, metagenome-assembled genomes, or single-cell genomes. Results are presented in the form of tables for metabolism and a variety of visualizations including biogeochemical cycling potential, representation of sequential metabolic transformations, community-scale microbial functional networks using a newly defined metric “MW-score” (metabolic weight score), and metabolic Sankey diagrams. METABOLIC takes ~ 3 h with 40 CPU threads to process ~ 100 genomes and corresponding metagenomic reads within which the most compute-demanding part of hmmsearch takes ~ 45 min, while it takes ~ 5 h to complete hmmsearch for ~ 3600 genomes. Tests of accuracy, robustness, and consistency suggest METABOLIC provides better performance compared to other software and online servers. To highlight the utility and versatility of METABOLIC, we demonstrate its capabilities on diverse metagenomic datasets from the marine subsurface, terrestrial subsurface, meadow soil, deep sea, freshwater lakes, wastewater, and the human gut.

**Conclusion:**

METABOLIC enables the consistent and reproducible study of microbial community ecology and biogeochemistry using a foundation of genome-informed microbial metabolism, and will advance the integration of uncultivated organisms into metabolic and biogeochemical models. METABOLIC is written in Perl and R and is freely available under GPLv3 at https://github.com/AnantharamanLab/METABOLIC.

Video abstract

**Supplementary Information:**

The online version contains supplementary material available at 10.1186/s40168-021-01213-8.

## Introduction

Metagenomics and single-cell genomics have transformed the field of microbial ecology by revealing a rich diversity of microorganisms from diverse settings, including terrestrial [1–3] and marine environments [4, 5] and the human body [6]. These approaches can provide an unbiased and insightful view into microorganisms mediating and contributing to biogeochemical activities at a number of scales ranging from individual organisms to communities [7–9]. Recent studies have also enabled the recovery of hundreds to thousands of genomes from a single sample or environment [8, 10, 11]. However, analyses of ever-increasing datasets remain a challenge. For example, there is a lack of scalable and reproducible bioinformatic approaches for characterizing metabolism and biogeochemistry, as well as standardizing their analyses and representation for large datasets.

Microbially mediated biogeochemical processes serve as important driving forces for the transformation and cycling of elements, energy, and matter among the lithosphere, atmosphere, hydrosphere, and biosphere [12]. Microbial communities in natural environmental settings exist in the form of complex and highly connected networks that share and compete for metabolites [13–15]. The interdependent and cross-linked metabolic and biogeochemical interactions within a community can provide a relatively high level of plasticity and flexibility [16]. For instance, multiple metabolic steps within a specific pathway are often separately distributed in a number of microorganisms and they are interdependent on utilizing the substrates from the previous step [2, 17, 18]. This scenario, referred to as “metabolic handoffs,” is based on sequential metabolic transformations, and provides the benefit of high resilience of metabolic activities which make both the community and function stable in the face of perturbations [17, 18]. It is therefore highly valuable to obtain the information of microbial metabolic function from the perspective of individual genomes as well as the entire microbial community.

Currently, there are many quantitative software and platforms for reconstructing species and community-level metabolic networks [19–25]. They are largely based on building microbial metabolic models containing reactions for substrate utilization and product generation [15, 19]. Based on individual microbial models, metabolic phenotypes for the whole community can be further predicted [15]. These approaches allow providing mechanistic bases for predicting and thus operating community metabolisms based on the given environmental conditions and predicted microbial phenotypes [26]. Thus, they are more focused on illustrating the operating principles of community metabolisms and the underlying metabolic networks of connected reactions to achieve better outcomes for metabolite production [21, 22], industrial applications [19], drug discovery [19], etc.

Yet, seldom have approaches been developed to study the functional role of microorganisms in the context of biogeochemistry and community-level functional networks [27, 28]. Such tools are based on the principles of facilitating the understanding of microbially mediated biogeochemical activities. The tools ask for identifying and providing metabolic predictions on the functional details, transformations of nutrients and energy, and functional connections for microorganisms within the community [29]. The resulting genome-informed microbial metabolisms are important for understanding the microbial roles within a whole community in mediating the biogeochemical processes. Currently, such quantitative approaches to interpret functional details, reconstruct metabolic relationships, and visualize microbial functional networks are still limited [27, 28].

Prediction of microbial metabolism relies on the annotation of protein function for microorganisms using a number of established databases, e.g., KEGG [30], MetaCyc [31], Pfam [32], TIGRfam [33], SEED/RAST [34], and eggNOG [35]. However, these results are often highly detailed, and therefore can be overwhelming to users. Obtaining a functional profile and identifying metabolic pathways in a microbial genome can involve manual inspection of thousands of genes [36]. Organizing, interpreting, and visualizing such datasets remains a challenge and is often untenable especially with datasets larger than one microbial genome. There is a critical need for approaches and tools to identify and validate the presence of metabolic pathways, biogeochemical function, and connections in microbial communities in a user-friendly manner. Such tools addressing this gap would also allow standardization of methods and easier integration of genome-informed metabolism into biogeochemical models, which currently rely primarily on physicochemical data and treat microorganisms as black boxes [37]. A recent statistical study indicates that incorporating microbial community structure in biogeochemical modeling could significantly increase model accuracy of processes that are mediated by narrow phylogenetic guilds via functional gene data, and processes that are mediated by facultative microorganisms via community diversity metrics [38]. This highlights the importance of integrating microbial community and genomic information into the prediction and modeling of biogeochemical processes.

Here, we present the software METABOLIC (METabolic And BiogeOchemistry anaLyses In miCrobes), a toolkit to profile metabolic and biogeochemical traits, and functional networks in microbial communities based on microbial genomes. METABOLIC integrates annotation of proteins using KEGG [30], TIGRfam [33], Pfam [32], custom hidden Markov model (HMM) databases [2], dbCAN2 [39], and MEROPS [40]; incorporates a protein motif validation step to accurately identify proteins based on prior biochemical validation; and determines the presence or absence of metabolic pathways based on KEGG modules. METABOLIC also produces user-friendly outputs in the form of tables and figures including a summary of microbial functional profiles, biogeochemically relevant pathways, functional networks at the scale of individual genomes and community levels, and microbial contributions to biogeochemical processes.

## Methods

### HMM databases used by METABOLIC

To generate a broad range of metabolic gene HMM profiles, we integrated three sets of HMM-based databases, which are KOfam [41] (July 2019 release, containing HMM profiles for KEGG/KO with predefined score thresholds), TIGRfam [33] (Release 15.0), Pfam [32] (Release 32.0), and custom metabolic HMM profiles [2]. In order to achieve a better HMM search result excluding non-specific hits, we have tested and manually curated cutoffs for those HMM databases listed above into the resulting HMMs: KOfam database—KOfam suggested values; TIGRfam/Pfam/Custom databases—manually curated by adjusting noise cutoffs (NC); or trusted cutoffs (TC) to avoid potential false positive hits. For the KOfam suggested cutoffs, we considered both the score type (full length or domain) and the score value to assign whether an individual protein hit is significant or not. HMM databases were used as the reference for hmmsearch [42] to find protein hits of input genomes. Prodigal [43] was used to annotate genomic sequences (the method used to find ORFs by Prodigal can be set by METABOLIC as “meta” or “single”), or a user can provide self-annotated proteins (with extensions of “.faa”) to facilitate incorporation into existing pipelines. Methods on the manual curation of these HMM databases are described in the next section.

### Curation of cutoff scores for metabolic HMMs

Two curation methods for adjusting NC or TC of TIGRfam/Pfam/Custom databases were used for a specific HMM profile. First, we parsed and downloaded representative protein sequences according to either the corresponding KEGG identifier or UniProt identifier [44]. We then randomly subsampled a small portion of the sequences (10% of the whole collection if this was more than 10 sequences, or at least 10 sequences) as the query to search against the representative protein collections [42]. Subsequently, we obtained a collection of hmmsearch scores by pairwise sequence comparisons. We plotted scores against hmmsearch hits and selected the mean value of the sharpest decreasing interval as the adjusted cutoff (approximately the F1 score). Second, we downloaded a collection of proteins that belong to a specific HMM profile and pre-checked the quality and phylogeny of these proteins by reconstructing and manually inspecting phylogenetic trees. We applied pre-checked protein sequences as the query search against a set of training metagenomes (data not shown). We then obtained a collection of hmmsearch scores of resulting hits from the training metagenomes. By using a similar method as described above, the cutoff was selected as the mean value of the sharpest decreasing interval.

The following example demonstrates how the method above was used to curate the cutoffs for hydrogenase enzymes. We then expanded this method to all genes using a similar method. We downloaded the individual protein collections for each hydrogenase functional group from the HydDB [45], which included [FeFe] Group A-C series, [Fe] Group, and [NiFe] Group 1–4 series. The individual hydrogenase functional groups were further categorized based on reaction directions, which included H_2_-evolution, H_2_-uptake, H_2_-sensing, electron-bifurcation, and bidirection. To define the NC cutoff (“--cut_nc” in hmmsearch) for individual hydrogenase groups, we used the protein sequences from each hydrogenase group as the query for hmmsearch against the overall hydrogenase collections. By plotting the resulting hmmsearch hit scores against individual hmmsearch hits, we selected the mean value of the sharpest decreasing interval as the cutoff value.

### Motif validation

To automatically validate protein hits and avoid false positives, we introduced a motif validation step by comparing protein motifs against a manually curated set of highly conserved residues in important proteins. This manually curated set of highly conserved residues is derived from either reported works or protein alignments from this study. We chose 20 proteins associated with important metabolisms (with a focus on important biogeochemical cycling steps) that are prone to be misannotated into proteins within the same protein family. Details of these proteins are provided in Additional file 8: Dataset S1. For example, DsrC (sulfite reductase subunit C) and TusE (tRNA 2-thiouridine synthesizing protein E) are similar proteins that are commonly misannotated. Both of them are assigned to the family KO:K11179 in the KEGG database. To avoid assigning TusE as a DsrC, we identified a specific motif for DsrC but not TusE (GPXKXXCXXXGXPXPXXCX”, where “X” stands for any amino acid) [46]. We used these specific motifs to filter out proteins that have high sequence similarity but functionally divergent homologs.

### Annotation of carbohydrate-active enzymes and peptidases

For carbohydrate-active enzymes (CAZymes), dbCAN2 [39] was used to annotate proteins with default settings. The hmmscan parser and HMM database (2019-09-05 release) were downloaded from the dbCAN2 online repository (http://bcb.unl.edu/dbCAN2/download/) [39]. The non-redundant library of protein sequences which contains all the peptidase/inhibitor units from the peptidase (inhibitor) database MEROPS [40] (known as the “MEROPS pepunit” database) was used as the reference database to search against putative peptidases and inhibitors using DIAMOND. The settings used for the DIAMOND BLASTP search were “-k 1 -e 1e-10 --query-cover 80 --id 50” [47]. We used the “MEROPS pepunit” database due to the fact that it only includes the functional unit of peptidases/inhibitors [40] which can effectively avoid potential non-specific hits.

### Implementation of METABOLIC-G and METABOLIC-C

To target specific applications in processing omics datasets, we have implemented two versions of METABOLIC: METABOLIC-G (genome version) and METABOLIC-C (community version). METABOLIC-G intakes only genome files and provides analyses for individual genome sequences (including three kinds of genomes, e.g., single-cell genomes, isolate genomes, and metagenome-assembled genomes). All analyses and procedures of METABOLIC-G for all these three kinds of genomes are identical.

METABOLIC-C includes an option for users to include metagenomic reads for mapping to metagenome-assembled genomes (MAGs). Using Bowtie 2 (version ≥ v2.3.4.1) [48], metagenomic BAM files were generated by mapping all input metagenomic reads to gene collections from input genomes. Subsequently, SAMtools (version ≥ v0.1.19) [49], BAMtools (version ≥ v2.4.0) [50], and CoverM (https://github.com/wwood/CoverM) were used to convert BAM files to sorted BAM files and to calculate the gene coverage. To calculate the relative abundance of a specific biogeochemical cycling step, all the coverage of genes that are responsible for this step were summed up and normalized by overall gene coverage. Reads from single-cell and isolate genomes can also be mapped in an identical manner to metagenomes. The gene coverage result generated by metagenomic read mapping was further used in downstream processing steps to conduct community-scale interaction and network analyses.

### Classifying microbial genomes into taxonomic groups

To study community-scale interactions and networks of each microbial group within the whole community, we classified microbial genomes into individual taxonomic groups. GTDB-Tk v0.1.3 [51] was used to assign taxonomy of input genomes with default settings. GTDB-Tk can provide automated and objective taxonomic classification based on the rank-normalized Genome Taxonomy Database (GTDB) taxonomy within which the taxonomy ranks were established by a sophisticated criterion counting the relative evolutionary divergence (RED) and average nucleotide identity (ANI) [51, 52]. Subsequently, genomes were clustered into microbial groups at the phylum level, except for Proteobacteria which were replaced by its subordinate classes due to its wide coverage. Taxonomic assignment information for each genome was used in the downstream community analyses.

### Analyses and visualization of metabolic outputs, biogeochemical cycles, MW-scores, functional networks, and metabolic Sankey diagrams

To visualize the outputted metabolic results, the R script “*draw_biogeochemical_cycles.R*” was used to draw the corresponding metabolic pathways for individual genomes. We integrated HMM profiles that are related to biogeochemical activities and assigned HMM profiles to 31 distinct biogeochemical cycling steps (See details in “METABOLIC_template_and_database” folder on the GitHub page). The script can generate figures showing biogeochemical cycles for individual genomes and the summarized biogeochemical cycle for the whole community. By using the results of metabolic profiling generated from hmmsearch and gene coverage from the mapping of metagenomic reads, we can depict metabolic capacities of both individual genomes and all genomes within a community as a whole. The community-level diagrams, including sequential transformation diagrams, functional network diagrams, and metabolic Sankey diagrams, were generated using both metabolic profiling and gene coverage results. The diagrams are made by the scripts “*draw_sequential_reaction_diagram.R*,” “*draw_metabolic_Sankey_diagram.R*,” and “*draw_functional_network_diagram.R*,” respectively (For details, refer to GitHub wiki pages).

MW-score (metabolic weight score) is a metric reflecting the functional capacity and abundance of a microbial community in co-sharing functional networks. It was calculated at the community-scale level based on results of metabolic profiling and gene coverage from metagenomic read mapping as described above. We divided metabolic/biogeochemical cycling steps (31 in total) into a finer level—function (51 functions in total)—for better resolution in reflecting functional networks. By using similar methods for determining metabolic interactions (as described above), we selected functions that are shared among genomes. MW-score for each function was calculated by summing up all the coverage values of each function (calculated by summing up all coverage values of genomes that contain this function) and subsequently normalizing it by the overall function coverage. For each function, the contribution percentage of each microbial phylum (the default taxonomic level setting) was also calculated accordingly. One can also change the taxonomic level setting to the resolution of “class,” “order,” “family,” or “genus” to calculate the corresponding contribution percentage of each microbial group. Two equations are provided as follows to calculate each function’s MW-score (1) and the percentage of contribution of each microbial group to the MW-score (2):

1 $${\mathrm{MW}}_{f_{\mathrm{i}}}=\frac{\sum_{g={g}_1}^{g_{\mathrm{n}}}{\mathrm{C}}_{g_{\mathrm{n}}}\cdot {\mathrm{S}}_{f_{\mathrm{i}}}}{\sum_{g={g}_1,f={f}_1}^{g_{\mathrm{n}},{f}_{\mathrm{n}}}{\mathrm{C}}_{g_{\mathrm{n}}}\cdot {\mathrm{S}}_{f_{\mathrm{n}}}}$$

2 $$\kern0.75em {\mathrm{C}\mathrm{perc}}_{f_{\mathrm{i}}{p}_{\mathrm{j}}}=\left(\frac{\sum_{g={g}_k}^{g_{\mathrm{l}}}{\mathrm{C}}_{g_{\mathrm{n}}}\cdot {\mathrm{S}}_{f_{\mathrm{i}}}}{\sum_{g={g}_1,f={f}_1}^{g_{\mathrm{n}},{f}_{\mathrm{n}}}{\mathrm{C}}_{g_{\mathrm{n}}}\cdot {\mathrm{S}}_{f_{\mathrm{n}}}}/\frac{\sum_{g={g}_1}^{g_{\mathrm{n}}}{\mathrm{C}}_{g_{\mathrm{n}}}\cdot {\mathrm{S}}_{f_{\mathrm{i}}}}{\sum_{g={g}_1,f={f}_1}^{g_{\mathrm{n}},{f}_{\mathrm{n}}}{\mathrm{C}}_{g_{\mathrm{n}}}\cdot {\mathrm{S}}_{f_{\mathrm{n}}}}\right)\times 100\%$$

within which *g*_*k*_…*g*_l_ ∈ *p*_j_

In Eq. (1), MW refers to MW-score. *f*_i_ refers to the studied function (*f*) which ranks in the (i) position among all functions. *g*_1_ and *g*_n_ indicate the first and the last genome among all genomes. *f*_1_ and *f*_n_ indicate the first and the last function among all functions. C_*g*_ means the coverage of a genome and S_*f*_ means the presence (denoted as 1) or absence (denoted as 0) state of a function within that genome. In Eq. (2), Cprec refers to the contribution percentage of a microbial group to the MW-score. *p*_j_ means the studied group (*p*) which ranks in the (j) position among all groups. *g*_k_ and *g*_l_ indicate the genomes which rank in the (k) position and the (l) position among all genomes; the additional note *g*_*k*_…*g*_l_ ∈ *p*_j_ indicates all the genomes between these two belong to the studied group *p*_j_.

### Example of METABOLIC analysis

An example of community-scale analyses including elemental biogeochemical cycling and sequential reaction analyses, functional network and metabolic Sankey visualization, and MW-score calculation were conducted using a metagenomic dataset of a microbial community inhabiting deep-sea hydrothermal vent environment of Guaymas Basin in the Pacific Ocean [53]. It contains 98 MAGs and 1 set of metagenomic reads (genomes were available at NCBI BioProject PRJNA522654 and metagenomic reads in NCBI SRA with accession as SRR3577362).

A metagenomic-based study of the microbial community from an aquifer adjacent to Colorado River, located near Rifle, has provided an accurate reconstruction of the metabolism and ecological roles of the microbial majority [2]. From underground water and sediments of the terrestrial subsurface at Rifle, 2545 reconstructed MAGs were obtained (genomes are under NCBI BioProject PRJNA288027). They were used as the in silico dataset to test METABOLIC’s performance. First, all the microbial genomes were dereplicated by dRep v2.0.5 [54] to pick the representative genomes for downstream analysis using the setting of “-comp 85.” Then, METABOLIC-G was applied to profile the functional traits of these representative genomes using default settings. Finally, the metabolic profile chart was depicted by assigning functional traits to GTDB taxonomy-clustered genome groups.

### Test of software performance across different environments

To benchmark and test the performance of METABOLIC in different environments, eight datasets of metagenomes and metagenomic reads from marine, terrestrial, and human environments were used. These included marine subsurface sediments [55] (Deep biosphere beneath Hydrate Ridge offshore Oregon), freshwater lake [56] (Lake Tanganyika, eastern Africa), colorectal cancer (CRC) patient gut [57], healthy human gut [57], deep-sea hydrothermal vent [53] (Guaymas Basin, Gulf of California), terrestrial subsurface sediments and water [2] (Rifle, CO, USA), meadow soils [58] (Angelo Coastal Range Reserve, CA, USA), and advanced water treatment facility [59] (Groundwater Replenishment System, Orange County, CA, USA). Default settings were used for running METABOLIC-C.

### Comparison of community-scale metabolism

To compare the metabolic profile of two environments at the community scale, MW-score was used as the benchmark. Two sets of environmental pairs were compared, including the pair of marine subsurface sediments [55] and terrestrial subsurface sediments [2] and the pair of freshwater lake [56] and deep-sea hydrothermal vent [53]. To demonstrate differences between these environments in specific biogeochemical processes, we focused on the biogeochemical cycling of sulfur. The sulfur biogeochemical cycling diagrams were depicted with the annotation of the number and the coverage of genomes that contain each biogeochemical cycling step.

### Metabolism in human microbiomes

To inspect the metabolism of microorganisms in the human microbiome (associated with skin, oral mucosa, conjunctiva, gastrointestinal tracts, etc.), a subset of KOfam HMMs (139 HMM profiles) were used as markers to depict the human microbiome metabolism (parsed by HuMiChip targeted functional gene families [60]). They included 10 function categories as follows: amino acid metabolism, carbohydrate metabolism, energy metabolism, glycan biosynthesis and metabolism, lipid metabolism, metabolism of cofactors and vitamins, metabolism of other amino acids, metabolism of terpenoids and polyketides, nucleotide metabolism, and translation. The CRC and healthy human gut (healthy control) sample datasets were used as the input (Accession IDs: BioProject PRJEB7774, Sample 31874, and Sample 532796). Heatmap of presence/absence of these functions were depicted by R package “*pheatmap*” [61] with 189 horizontal entries (there are duplications of HMM profiles among function categories; for detailed human microbiome metabolism markers, refer to Additional file 9: Dataset S2).

### Representation of microbial cell metabolism

To provide a schematic representation of the metabolism of microbial cells, two microbial genomes were used as examples, Hadesarchaea archaeon 1244-C3-H4-B1 and Nitrospirae bacteria M_DeepCast_50m_m2_151. METABOLIC-G results of these two genomes, including functional traits and KEGG modules, were used to draw the cell metabolism diagrams.

### Metatranscriptome analysis by METABOLIC

METABOLIC-C can take metatranscriptomic reads as input into transcript coverage calculation and integrate the result into downstream community analyses. METABOLIC-C uses a similar method to that of gene coverage calculation, including mapping transcriptomic reads to the gene collection from input genomes, converting BAM files to sorted BAM files, and calculating the transcript coverage. The raw transcript coverage was further normalized by the gene length and metatranscriptomic read number in Reads Per Kilobase of transcript, per Million mapped reads (RPKM). Hydrothermal vent and background seawater transcriptomic reads from Guaymas Basin (NCBI SRA accessions: SRR452448 and SRR453184) were used to test the outcome of metatranscriptome analysis.

## Results

Given the ever-increasing number of microbial genomes from microbiome studies, we developed METABOLIC to enable metabolic pathway analysis and visualization of biogeochemical cycles and community-scale functional networks. METABOLIC has an improved methodology to get fast, accurate, and robust annotation results, and it integrates a variety of visualization functions for better interpreting community-level functional interactions and microbial contributions. While METABOLIC relies on microbial genomes and metagenomic reads for underpinning its analyses for community-level functional interactions, it can easily integrate transcriptomic datasets to provide an activity-based measure of community networks. The scalable capacity, wide utility, and compatibility for analyzing datasets from various environments make it a well-tailored tool for metabolic profiling of large sets of genomes. In the following sections, the microbial community consisting of 98 MAGs from a deep-sea hydrothermal vent was used as the input dataset if not mentioned otherwise.

### Workflow to determine the presence of metabolic pathways

**Fig. 1:**
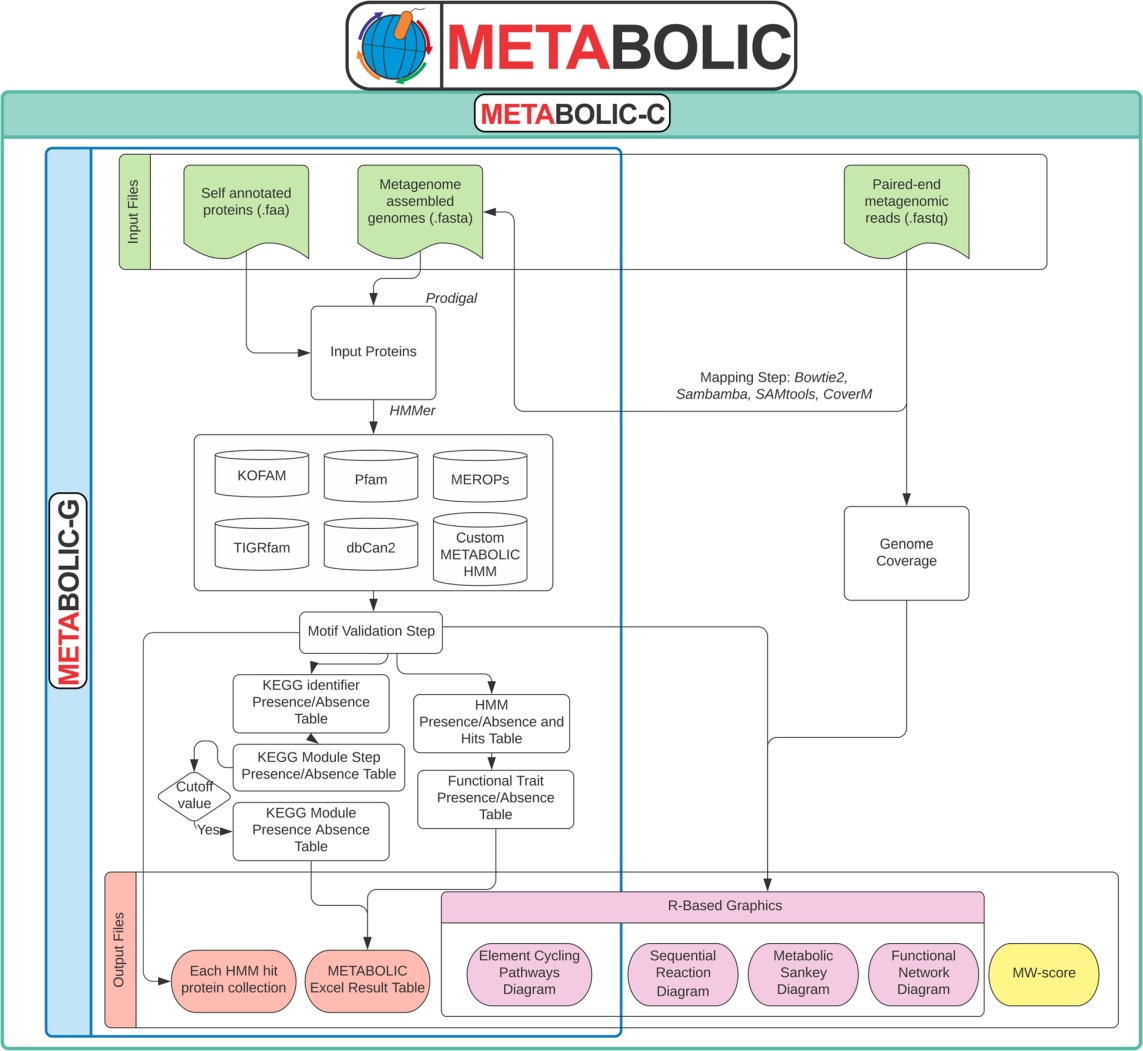
An outline of the workflow of METABOLIC. Detailed instructions are available at https://github.com/AnantharamanLab/METABOLIC/wiki. METABOLIC-G workflow is specifically shown in the blue box and METABOLC-C workflow is shown in the green square

METABOLIC is written in Perl and R and is expected to run on Unix, Linux, or macOS. The prerequisites are described on METABOLIC’s GitHub wiki pages (https://github.com/AnantharamanLab/METABOLIC/wiki). The input folder requires microbial genome sequences in FASTA format and an optional set of genomic/metagenomic reads which were used to reconstruct those genomes (Fig. 1). The annotated proteins from input genomic sequences are queried against HMM databases (KEGG KOfam, Pfam, TIGRfam, and custom HMMs) using hmmsearch implemented within HMMER [42] which applies methods to detect remote homologs as sensitively and efficiently as possible. After the hmmsearch step, METABOLIC subsequently validates the primary outputs by a motif-checking step for a subset of protein families; only those protein hits which successfully pass this step are regarded as positive hits.

METABOLIC relies on matches to the above databases to infer the presence of specific metabolic pathways in microbial genomes. Individual KEGG annotations are inferred in the context of KEGG modules for a better interpretation of metabolic pathways. A KEGG module is comprised of multiple steps with each step representing a distinct metabolic function. We parsed the KEGG module database [62] to link the existing relationship of KO identifiers to KEGG module identifiers to project our KEGG annotation result into the interactive network which was constructed by individual building blocks—modules—for better representation of metabolic blueprints of input genomes. In most cases, we used KOfam HMM profiles for KEGG module assignments. For a specific set of important metabolic marker proteins and commonly misannotated proteins, we also applied the TIGRfam/Pfam/custom HMM profiles and motif-validation steps. The software has customizable settings for increasing or decreasing the priority of specific databases, primarily meant to increase annotation confidence by preferentially using custom HMM databases over KEGG KOfam when both targeting the same set of proteins.

Since individual genomes from metagenomes and single-cell genomes can often have incomplete metabolic pathways due to their low completeness compared to isolate genomes, we provide an option to determine the completeness of a metabolic pathway (or a module here). A user-defined cutoff is used to set the threshold of completeness for a given module to be assigned as present (the default cutoff is the presence of 75% of metabolic steps/genes within a given module), which is then used to produce a KEGG module presence/absence table. All modules exceeding the cutoff value are determined to be present. Meanwhile, the presence/absence information for each module step is also summarized in an overall output table to facilitate further detailed investigations.

Outputs consist of six different results that are reported in an Excel spreadsheet (Additional file 1: Fig. S1). These contain details of protein hits (Additional file 1: Fig. S1A) which include both presence/absence and protein names, presence/absence of functional traits (Additional file 1: Fig.S1B), presence/absence of KEGG modules (Additional file 1: Fig. S1C), presence/absence of KEGG module steps (Additional file 1: Fig. S1D), carbohydrate-active enzyme (CAZyme) hits (Additional file 1: Fig. S1E), and peptidase/inhibitor hits (Additional file 1: Fig. S1F). For each HMM profile, the protein hits from all input genomes can be used to construct phylogenetic trees or further be combined with reference protein collections for detailed evolutionary analyses.

### Quantitative visualization of biogeochemical cycles and sequential reactions

**Fig. 2:**
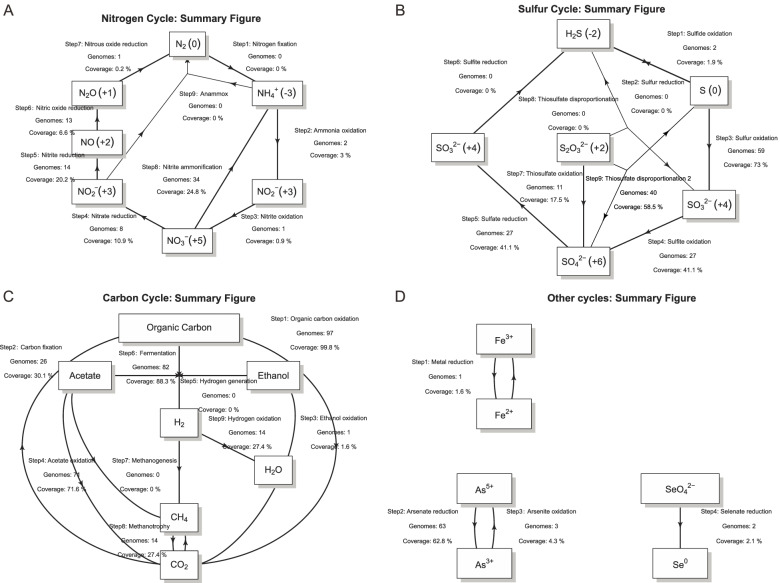
Summary scheme of biogeochemical cycling processes at the community scale. Each arrow represents a single transformation/step within a cycle. Labels above each arrow are (from top to bottom): step number and reaction, number of genomes that can conduct these reactions, metagenomic coverage of genomes (represented as a percentage within the community) that can conduct these reactions. The numbers in brackets next to the nitrogen or sulfur-containing compounds are chemical states of the nitrogen or sulfur atoms in these compounds

After METABOLIC generates protein and pathway annotation results, the software further identifies and highlights specific pathways of importance in microbiomes associated with energy metabolism and biogeochemistry. To visualize pathways of biogeochemical importance, it generates schematic profiles for nitrogen, carbon, sulfur, and other elemental cycles for each genome. The set of genomes used as input is considered the “community,” and each genome within is considered an “organism.” A summary schematic diagram at the community level integrates results from all individual genomes within a given dataset (Fig. 2) and includes computed abundances for each step in a biogeochemical cycle if the genomic/metagenomic read datasets are provided. The genome number labeled in the figure indicates the number/quantity of genomes that contain the specific gene components of a biogeochemical cycling step (Fig. 2) [2]. In other words, it represents the number of organisms within a given community inferred to be able to perform a given metabolic or biogeochemical transformation. The abundance percentage indicates the relative abundance of microbial genomes that contain the specific gene components of a biogeochemical cycling step among all microbial genomes in a given community (Fig. 2) [2].

**Fig. 3:**
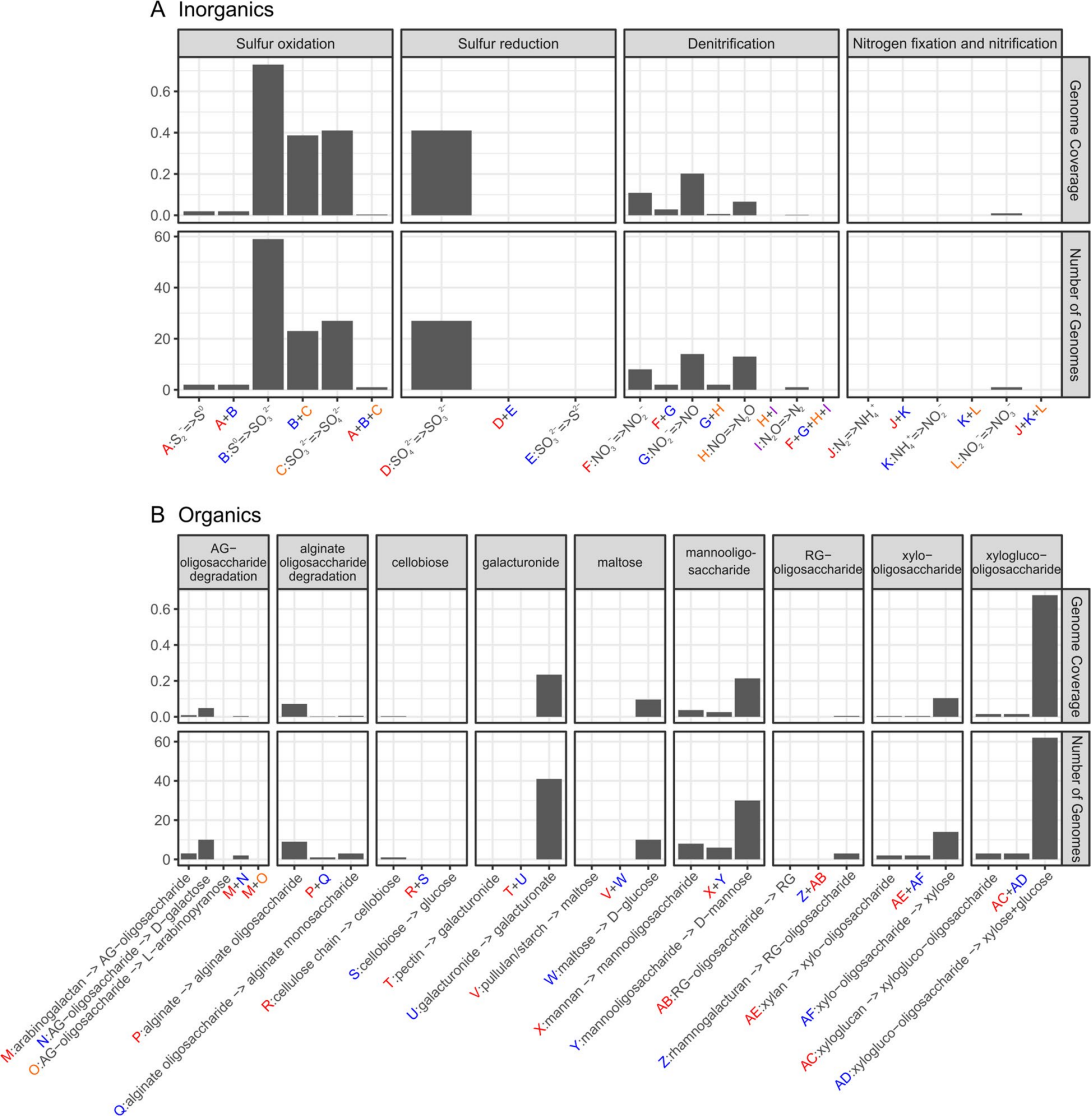
Schematic figure of sequential metabolic transformations. **A** The sequential transformation of inorganic compounds. **B** The sequential transformation of organic compounds. *X*-axes describe individual sequential transformations indicated by letters. The two panels describe the number of genomes and genome coverage (represented as a percentage within the community) of organisms that are involved in certain sequential metabolic transformations. The deep-sea hydrothermal vent dataset was used for these analyses

Microorganisms in nature often do not encode pathways for the complete transformation of compounds. For example, microorganisms possess partial pathways for denitrification that can release intermediate compounds like nitrite, nitric oxide, and nitrous oxide in lieu of nitrogen gas which is produced by complete denitrification [63]. A greater energy yield could be achieved if one microorganism conducts all steps associated with a pathway (such as denitrification) [2] since it could fully use all available energy from the reaction. However, in reality, few organisms in microbial communities carry out multiple steps in complex pathways; organisms commonly rely on other members of microbial communities to conduct sequential reactions in pathways [2, 64, 65]. Thus, to study this metabolic scenario in microbial communities, METABOLIC summarizes and enables visualization of the genome number and coverage (relative abundance) of microorganisms that are putatively involved in the sequential transformation of both important inorganic and organic compounds (Fig. 3). This provides a quantitative calculation of microbial interactions and connections using shared metabolites associated with inorganic and organic transformations. Additionally, it shows the intuitive pattern of quantity and abundance of microorganisms that are able to conduct partial or all steps for a given pathway, which potentially reflects the degree of resilience of a microbial community.

### Calculation and visualization of functional networks, metabolic weight scores (MW-scores), and microbial contribution to metabolic reactions

**Fig. 4:**
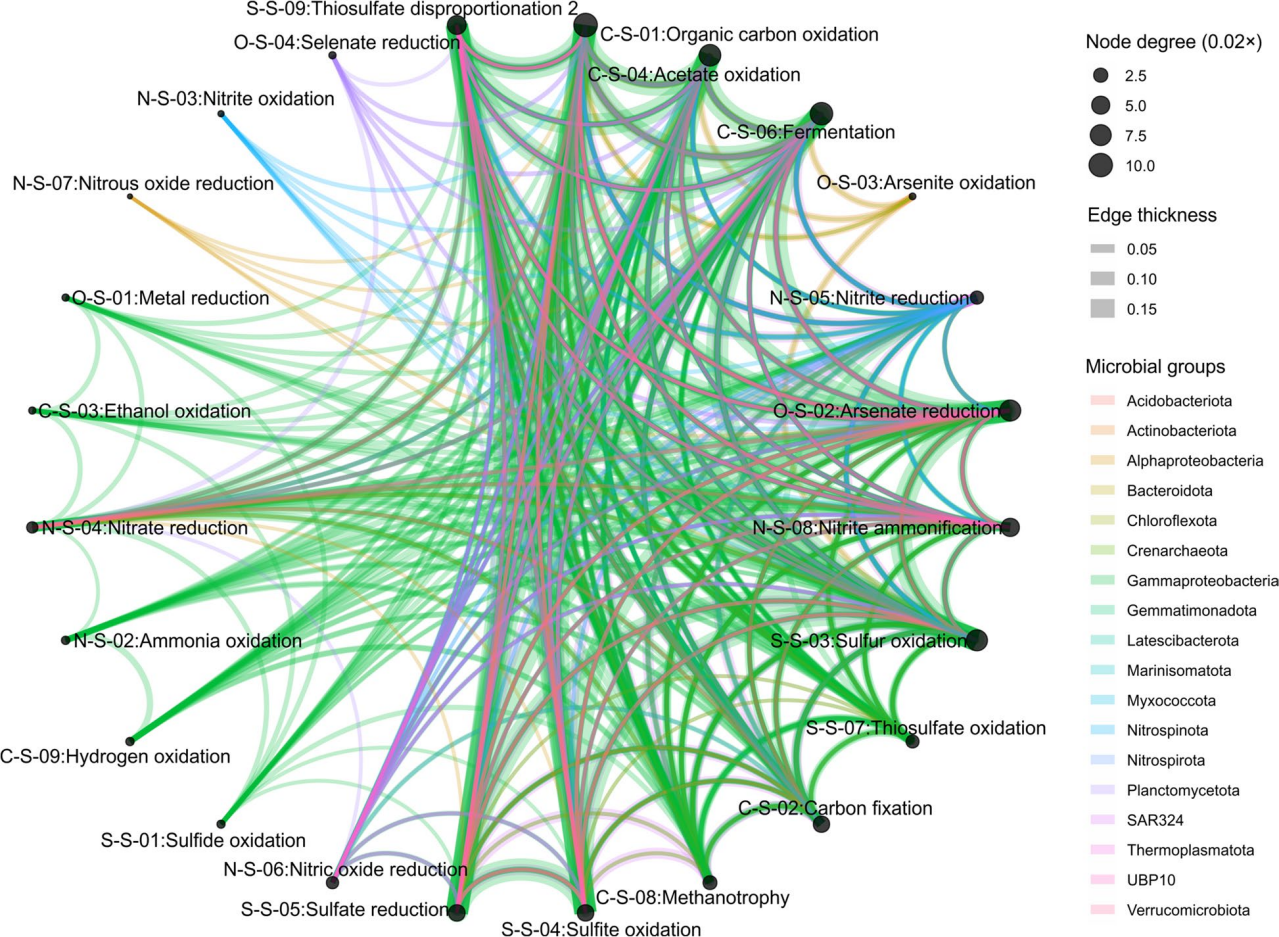
Functional network showing connections between different functions in the microbial community. Nodes represent individual steps in biogeochemical cycles; edges connecting two given nodes represent the functional connections between nodes, which are enabled by organisms that can conduct both biogeochemical processes/steps. The size of the node was depicted according to the degree (number of connections to each node). The thickness of the edge was depicted according to the average gene coverage values of the two connected biogeochemical cycling steps—for example, thiosulfate oxidation and organic carbon oxidation. The color of the edges was assigned based on the taxonomy of the represented genome. The deep-sea hydrothermal vent dataset was used for these analyses

Given the microbial pathway abundance information generated by METABOLIC, we identified co-existing metabolisms in microbial genomes as a measure of connections between different metabolic functions and biogeochemical steps. In the context of biogeochemistry, this approach allows the evaluation of relatedness among biogeochemical steps and the connection contribution by microorganisms. This is enabled at the resolution of individual microbial groups based on the phylogenetic classification (Fig. 4) assigned by GTDB-Tk [51]. As an example, we have demonstrated this approach on a microbial community inhabiting deep-sea hydrothermal vents. We divided the microbial community of deep-sea hydrothermal vents into 18 phylum-level groups (except for Proteobacteria which were divided into their subordinate classes). The functional network diagrams were depicted at the resolution of both individual phyla and the entire community level (Additional file 10: Dataset S3). Figure 4 demonstrates metabolic connections that were represented with individual metabolic/biogeochemical cycling steps depicted as nodes, and the connections between two given nodes depicted as edges. The size of a given node is proportional to the degree (number of connections to each node). The thickness of a given edge was depicted based on the average of gene coverage values of two biogeochemical cycling steps (the connected nodes). More edges connecting two nodes represent more connections between these two steps. The color of the edge corresponds to the taxonomic group. At the whole community level, more abundant microbial groups were more represented in the diagram (Fig. 4). Overall, METABOLIC provides a comprehensive approach to construct and visualize functional networks associated with important pathways of energy metabolism and biogeochemical cycles in microbial communities and ecosystems.

**Fig. 5:**
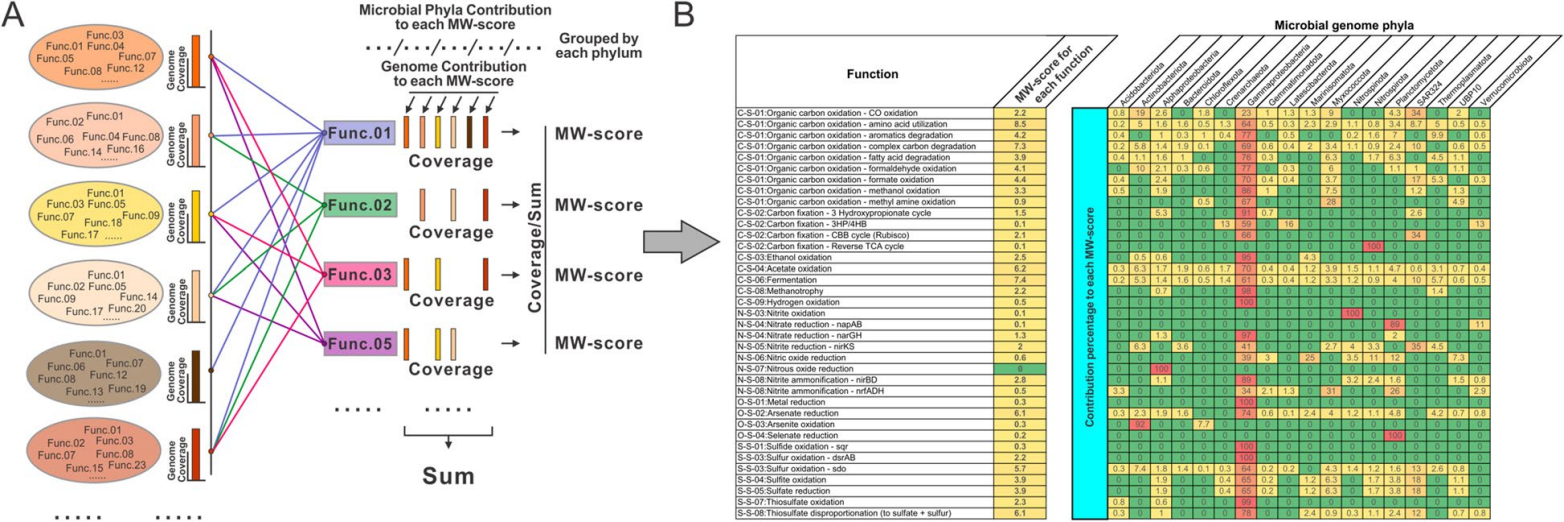
Description, calculation, and result table of MW-scores. **A** The calculation method for MW-score within a community based on a given metagenomic dataset. Each circle stands for a genome within the community, and the adjacent bar stands for its genome coverage within the community. The coverage values of encoded genes for all functions were summed up as the denominator, and the coverage value of encoded genes for each function was used as the numerator, and MW-score was calculated accordingly for each function. **B** The resulting table of MW-score for the deep-sea hydrothermal vent metagenomic dataset. MW-score for each function was given in a separated column, and the rest of the table indicates the contribution percentage to each MW-score of the genomes grouped in each phylum. The MW-score of “N-S-07:Nitrous oxide reduction” was not exactly 0 but rounded to 0 due to the original number being less than 0.05. Additionally, contribution percentages were also rounded to only retain one digit after the decimal points; consequently, the sum contribution percentages for some functions slightly deviate from 100%

To address the lack of quantitative and reproducible measures to represent potential metabolic interactions in microbial communities, we developed a new metric that we termed MW-score (metabolic weight scores) (Eqs. 1 and 2). MW-scores quantitatively measure “function weights” within a microbial community as reflected by the metabolic profile and gene coverage. As metabolic potential for the whole community was profiled into individual functions that either mediated specific pathways or transformed certain substrates into products, a function weight that reflects the abundance fraction for each function can be used to represent the overall metabolic potential of the community. MW-scores resolved the functional capacity and abundance in the co-sharing functional networks as studied and visualized in the above section. More frequently shared functions and their higher abundances lead to higher MW-scores, which quantitatively reflects the function weights in functional networks (Fig. 5). MW-score reflects the same functional networking pattern as the above description on the edges (networking lines) connecting the nodes (metabolic steps) that—more edges connecting two nodes indicates two steps are more shared, thicker edges indicate higher gene abundance for the metabolic steps. MW-scores can integratively represent these two networking patterns and serve as metrics to measure these function weights. At the same time, we also calculated each microbial group’s (phylum in this case) contribution to the MW-score of a specific function within the community (Fig. 5). A higher microbial group contribution percentage value indicates that one function is more represented by the microbial group (for both gene presence and abundance) in the functional networks. MW-scores provide a quantitative measure of comparing function weights and microbial group contributions within functional networks.

**Fig. 6:**
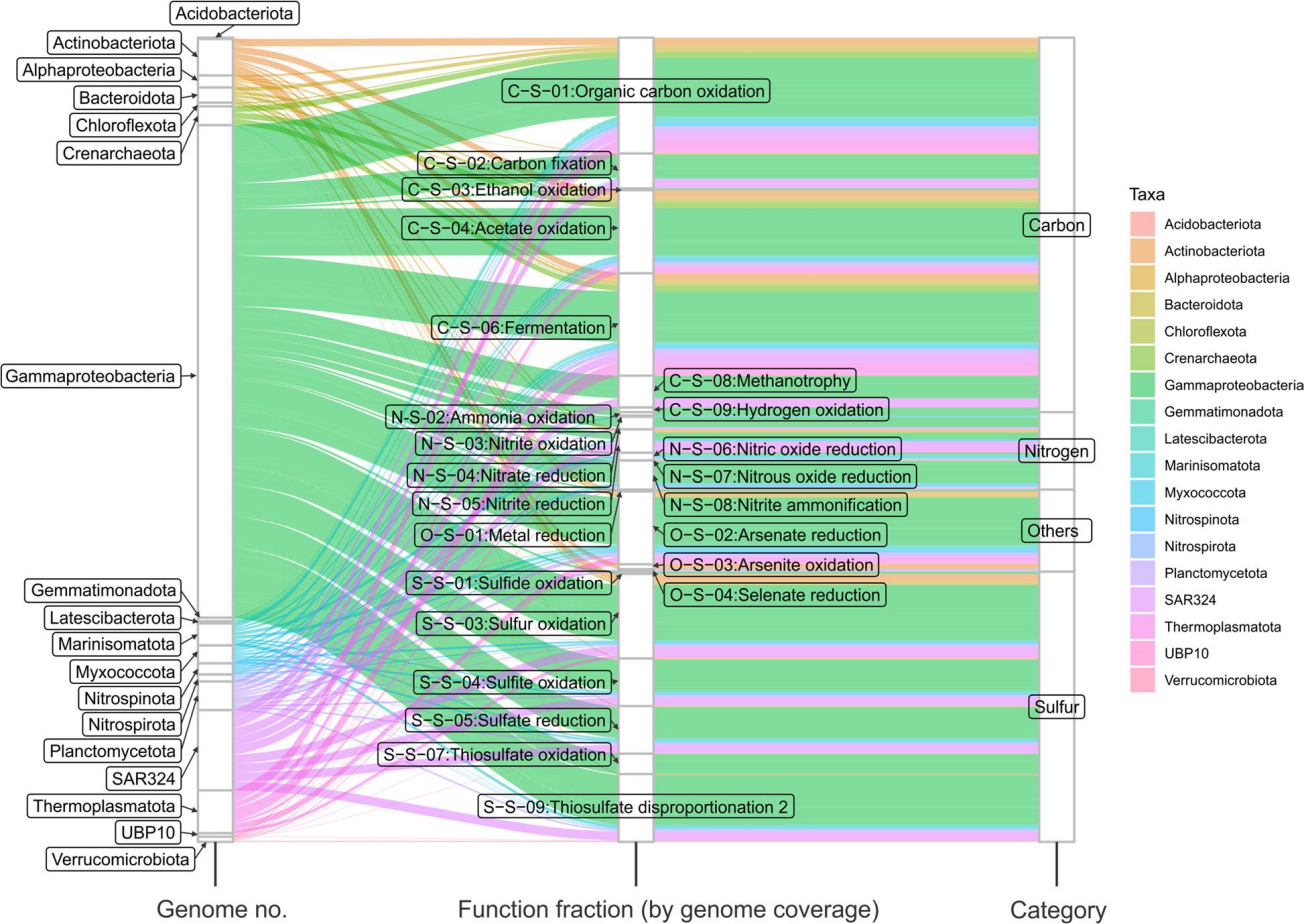
Metabolic Sankey diagram representing the contributions of microbial genomes to individual metabolic and biogeochemical processes, and entire elemental cycles. Microbial genomes are represented at the phylum-level resolution. The three columns from left to right represent taxonomic groups scaled by the number of genomes, the contribution to each metabolic function by microbial groups calculated based on genome coverage, and the contribution to each functional category/biogeochemical cycle. The colors were assigned based on the taxonomy of the microbial groups. The deep-sea hydrothermal vent dataset was used for these analyses

To understand the contributions of microbial groups associated with specific metabolic and biogeochemical transformations, we developed an approach to visualize the connections among specific taxonomic groups, metabolic reactions, and entire biogeochemical cycles such as carbon, nitrogen, and sulfur cycles. Our approach involves the use of Sankey diagrams (also called “*Alluvial*” plots) to represent the fractions of metabolic functions that are contributed by various microbial groups in a given community (Fig. 6). It allows visualization of metabolic reactions as the link between microbial contributors clustered as taxonomic groups and biogeochemical cycles at a community level (Fig. 6 and Additional file 10: Dataset S3). The function fraction was calculated by accumulating the genome coverage values of genomes from a specific microbial group that possesses a given functional trait. The width of curved lines from a specific microbial group to a given functional trait indicates their corresponding proportional contribution to a specific metabolism (Fig. 6). Alternatively, the genomic/metagenomic datasets which are used in constructing the above two diagrams: functional network diagram (Fig. 4) and metabolic Sankey diagram (Fig. 6), can be replaced by transcriptomic/metatranscriptomic datasets, and correspondingly, the gene coverage values will be replaced by gene expression values, and therefore, diagrams will represent the transcriptional activity patterns of functional network and microbial contribution to metabolic reactions (Additional file 2, 3, 4, and 5: Figure S2, S3, S4, and S5).

To demonstrate this part of the workflow, the microbial community consisting of 98 MAGs from a deep-sea hydrothermal vent was used as a test dataset. After running the bioinformatic analyses described above, resulting tables and diagrams were compiled and visualized accordingly (Figs. 4, 5, 6, and Additional file 10: Dataset S3). Results for functional networks and MW-scores of the deep-sea hydrothermal vent environment indicate that the microbial community depends on mixotrophy and sulfur oxidation for energy conservation and involves arsenate reduction potentially responsible for detoxification/arsenate resistance [66]. MW-scores indicate that amino acid utilization, complex carbon degradation, acetate oxidation, and fermentation are the major heterotrophic metabolisms for this environment; CO_2_-fixation and sulfur oxidation also occupy a considerable functional fraction, which indicates heterotrophy and autotrophy both contribute to energy conservation (Fig. 5). As represented by both MW-scores and metabolic Sankey diagram, Gammaproteobacteria are the most numerically abundant group in the community and they occupy significant functional fractions among both heterotrophic and autotrophic metabolisms (MW-score contribution ranging from 59-100%) (Figs. 5 and 6), which is consistent with previous findings in the Guaymas Basin hydrothermal environment [53, 67]. Meanwhile, MW-scores also explicitly reflect the involvement of other minor electron donors in energy conservation which are mainly contributed by Gammaproteobacteria, such as hydrogen and methane (Fig. 5). This is also consistent with previous findings [53, 67] and indicates the accuracy and sensitivity of MW-scores to reflect metabolic potentials.

### METABOLIC performance demonstration

To test METABOLIC’s performance on speed, we applied the software (METABOLIC-C mode) to analyze the metagenomic dataset which includes 98 MAGs from a deep-sea hydrothermal vent, and two sets of metagenomic reads (that are subsets of original reads with 10 million reads for each pair comprising ~ 10% of the total reads). The total running time was ~ 3 h using 40 CPU threads in a Linux version 4.15.0-48-generic server (Ubuntu v5.4.0). The most compute-demanding step is hmmsearch, which took ~ 45 min. When tested on another dataset comprising ~ 3600 microbial genomes (data not shown), METABOLIC could complete hmmsearch in ~ 5 h by using 40 CPU threads, indicating its scalable capability on analyzing thousands of genomes.

In order to test the accuracy of the results predicted by METABOLIC, we picked 15 bacterial and archaeal genomes from Chloroflexi, Thaumarchaeota, and Crenarchaeota which are reported to have 3 hydroxypropionate cycle (3HP) and/or 3-hydroxypropionate/4-hydroxybutyrate cycle (3HP/4HB) for carbon fixation. METABOLIC predicted results in line with annotations from the KEGG genome database which can be visualized in KEGG Mapper (Table 1). Our predictions are also in accord with biochemical evidence of the existence of corresponding carbon fixation pathways in each microbial group: (1) 3 out of 5 *Chloroflexi* genomes are predicted by both METABOLIC and KEGG to possess the 3HP pathway and none of all these *Chloroflexi* genomes are predicted to possess the 3HP/4HB pathway. This is consistent with current reports based on biochemical and molecular experiments that only organisms from the phylum *Chloroflexi* are known to possess the 3HP pathway [68] (Table 1). (2) All 5 *Thaumarchaeota* genomes and 2 out of 5 *Crenarchaeota* genomes are predicted by both METABOLIC and KEGG to possess the 3HP/4HB pathway and none of these *Thaumarchaeota* and *Crenarchaeota* genomes are predicted to possess the 3HP pathway. This is consistent with current reports that only the 3HP/4HB pathway could be detected in *Crenarchaeota* and *Thaumarchaeota* [69, 70] (Table 1). We also applied METABOLIC on a large well-studied dataset comprising 2545 metagenome-assembled genomes from terrestrial subsurface sediments and groundwater [2]. The annotation results of METABOLIC are consistent with previously described reports (Additional file 6, 10: Fig. S6, Dataset S3). These results suggest that METABOLIC can provide accurate annotations and perform well as a functional predictor for microbial genomes and communities.

**Table 1:** The carbon fixation metabolic traits of 15 tested bacterial and archaeal genomes predicted by both METABOLIC and KEGG genome database

				METABOLIC result		KEGG genome pathway	
				Carbon fixation		Carbon fixation	
Accession ID	Organism	KEGG Organism Code	Group	3HP cycle	3HP/4HB cycle	3HP cycle	3HP/4HB cycle
GCA_000011905.1	*Dehalococcoides mccartyi* 195	det	Chloroflexi	Absent	Absent	Absent	Absent
GCA_000017805.1	*Roseiflexus castenholzii* DSM 13941	rca	Chloroflexi	Present	Absent	Present	Absent
GCA_000018865.1	*Chloroflexus aurantiacus* J-10-fl	cau	Chloroflexi	Present	Absent	Present	Absent
GCA_000021685.1	*Thermomicrobium roseum* DSM 5159	tro	Chloroflexi	Absent	Absent	Absent	Absent
GCA_000021945.1	*Chloroflexus aggregans* DSM 9485	cag	Chloroflexi	Present	Absent	Present	Absent
GCA_000299395.1	*Nitrosopumilus sediminis* AR2	nir	Thaumarchaeota	Absent	Present	Absent	Present
GCA_000698785.1	*Nitrososphaera viennensis* EN76	nvn	Thaumarchaeota	Absent	Present	Absent	Present
GCA_000875775.1	*Nitrosopumilus piranensis* D3C	nid	Thaumarchaeota	Absent	Present	Absent	Present
GCA_000812185.1	*Nitrosopelagicus brevis* CN25	nbv	Thaumarchaeota	Absent	Present	Absent	Present
GCA_900696045.1	*Nitrosocosmicus franklandus* NFRAN1	nfn	Thaumarchaeota	Absent	Present	Absent	Present
GCA_000015145.1	*Hyperthermus butylicus* DSM 5456	hbu	Crenarchaeota	Absent	Absent	Absent	Absent
GCA_000017945.1	*Caldisphaera lagunensis* DSM 15908	clg	Crenarchaeota	Absent	Present	Absent	Present
GCA_000148385.1	*Vulcanisaeta distributa* DSM 14429	vdi	Crenarchaeota	Absent	Absent	Absent	Absent
GCA_000193375.1	*Thermoproteus uzoniensis* 768-20	tuz	Crenarchaeota	Absent	Present	Absent	Present
GCA_003431325.1	*Acidilobus* sp. 7A	acia	Crenarchaeota	Absent	Absent	Absent	Absent

**Fig. 7:**
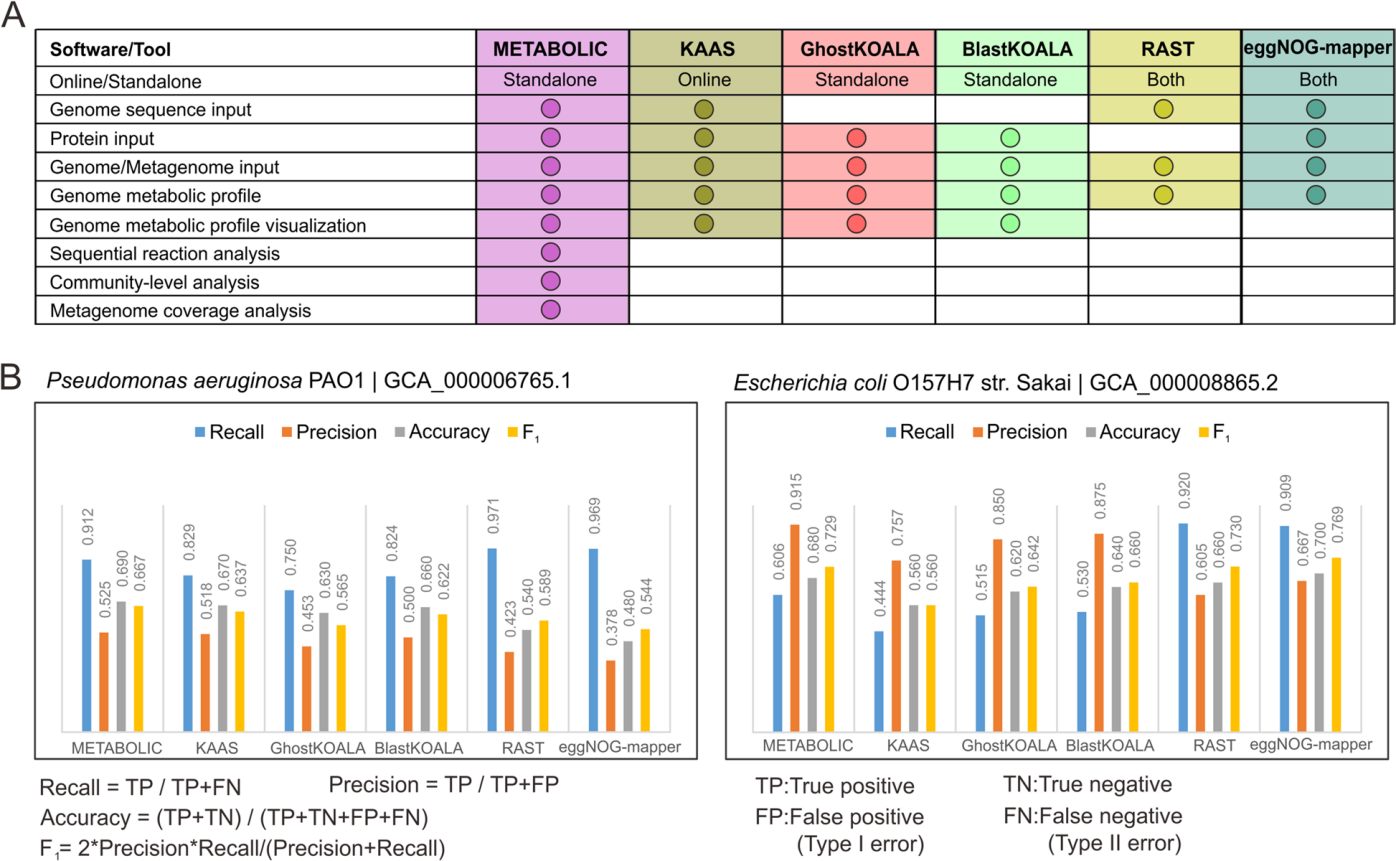
Comparison of METABOLIC with other software packages and online servers. **A** Comparison of workflows and services. **B** Comparison of performance of protein prediction for two representative genomes, *Pseudomonas aeruginosa* PAO1, and *Escherichia coli* O157H7 str. sakai

Currently, several software packages and online servers are available for genome annotation and metabolic profiling. Compared to other software/online servers including GhostKOALA [71], BlastKOALA [71], KAAS [72], RAST/SEED [34], and eggNOG-mapper [73], METABOLIC is unique in its ability to integrate multi-omic information toward elucidating and visualizing community-level functional connections and the contribution of microorganisms to biogeochemical cycles (Fig. 7A). Additionally, in order to compare the prediction performance of METABOLIC to others, we conducted parallel in silico experiments (Fig. 7B). We used two representative bacterial genomes as the test datasets. We randomly picked 100 protein sequences from individual genomes and submitted them to annotation by these six software/online servers. Predicted protein annotations by individual software and online servers were compared to their original annotations that were provided by the NCBI database (Additional file 11, 12: Dataset S4, S5). According to statistical methods of evaluating binary classification [74], the following parameters were used to make the comparison: (1) recall (also referred to as sensitivity) as the true positive rate, (2) precision (also referred to as the positive predictive value) which indicates the reproducibility and repeatability of a measurement system, (3) accuracy which indicates the closeness of measurements to their true values, and (4) *F*_1_ value which is the harmonic mean of precision and recall, and reflects both these two parameters. Among the tested software/online servers, the performance parameters of METABOLIC consistently placed it amongst the top 3 and top 2 software for recall and *F*_1_ and the top 1 and top 2 software for precision and accuracy. These results demonstrate that METABOLIC (Fig. 7B) provides robust performance and consistent metabolic prediction that facilitate accurate and reliable applicability for downstream data visualization and community-level analyses.

**Fig. 8:**
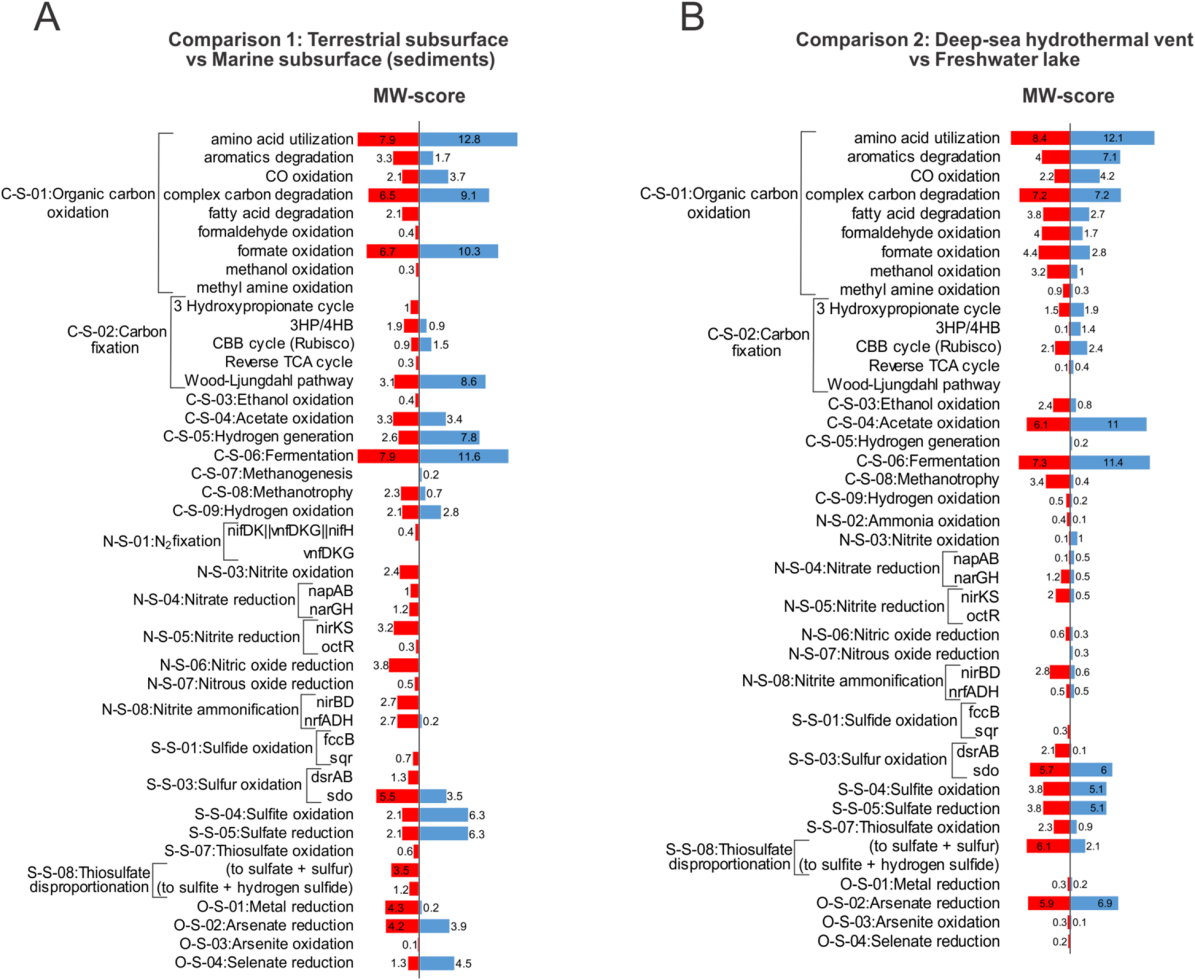
Community metabolism comparison based on MW-scores. **A** Comparison between terrestrial subsurface (left red bars) and marine subsurface (right blue bars). **B** Comparison between deep-sea hydrothermal vent (left red bars) and freshwater lake (right blue bars). MW-scores were calculated as gene coverage fractions for individual metabolic functions. Functions with MW-scores in both environments as zero were removed from each panel, e.g., N-S-02:Ammonia oxidation, N-S-09:Anammox, S-S-02:Sulfur reduction, and S-S-06:Sulfite reduction in panel (**A**), and C-S-07:Methanogenesis, N-S-01:N_2_ fixation, N-S-09:Anammox, S-S-02:Sulfur reduction, and S-S-06:Sulfite reduction in panel (**B**). For details for MW-score and each microbial group contribution, refer to Supplementary Dataset S3

To demonstrate the application and performance of METABOLIC in different samples, we tested eight distinct environments (marine subsurface, terrestrial subsurface, deep-sea hydrothermal vent, freshwater lake, gut microbiome from patients with colorectal cancer, gut microbiome from healthy control, meadow soil, wastewater treatment facility). Overall, we found METABOLIC to perform well across all the environments to profile microbial genomes with functional traits and biogeochemical cycles (Additional file 10: Dataset S3). Among these tested environments, we also performed community-scale metabolic comparisons based on the MW-score (Fig. 8). MW-score at the community scale reflects the overall metabolic profile distribution patterns. Specifically, we compared samples from terrestrial and marine subsurface and samples from hydrothermal vent and freshwater lake. We observed that terrestrial subsurface contains more abundant metabolic functions related to nitrogen cycling compared to the marine subsurface (Fig. 8A), consistent with the previous characterization of these two environments [2, 75]. Deep-sea hydrothermal vent samples had a considerably high concentration of methane and hydrogen [53] as compared to Lake Tanganyika (freshwater lake). Consistent with this phenomenon, the deep-sea hydrothermal vent microbial community has more abundant metabolic functions associated with methanotrophy and hydrogen oxidation (Fig. 8B). In order to focus on a specific biogeochemical cycle, we applied METABOLIC to compare sulfur-related metabolisms at the community scale for these two environment pairs (Additional file 7: Fig. S7). Terrestrial subsurface contains genomes covering more sulfur cycling steps compared to marine subsurface (7 steps vs. 3 steps) (Additional file 7: Fig. S7A). Freshwater lake contains genomes involving almost all the sulfur cycling steps except for sulfur reduction, while deep-sea hydrothermal vent contains less sulfur cycling steps (8 steps vs. 6 steps) (Additional file 7: Fig. S7B). Nevertheless, deep-sea hydrothermal vent has a higher fraction of genomes (59/98) and a higher relative abundance (73%) of these genomes involving sulfur oxidation compared to the freshwater lake (Additional file 7: Fig. S7B). This indicates that the deep-sea hydrothermal vent microbial community contains sulfur metabolism biased toward sulfur oxidation, which is consistent with previous metabolic characterization on the dependency of elemental sulfur in this environment [53, 76–78]. Collectively, by characterizing community-scale metabolism, METABOLIC can facilitate the comparison of overall functional profiles as well as for a particular elemental cycle.

### METABOLIC enables accurate reconstruction of cell metabolism

**Fig. 9:**
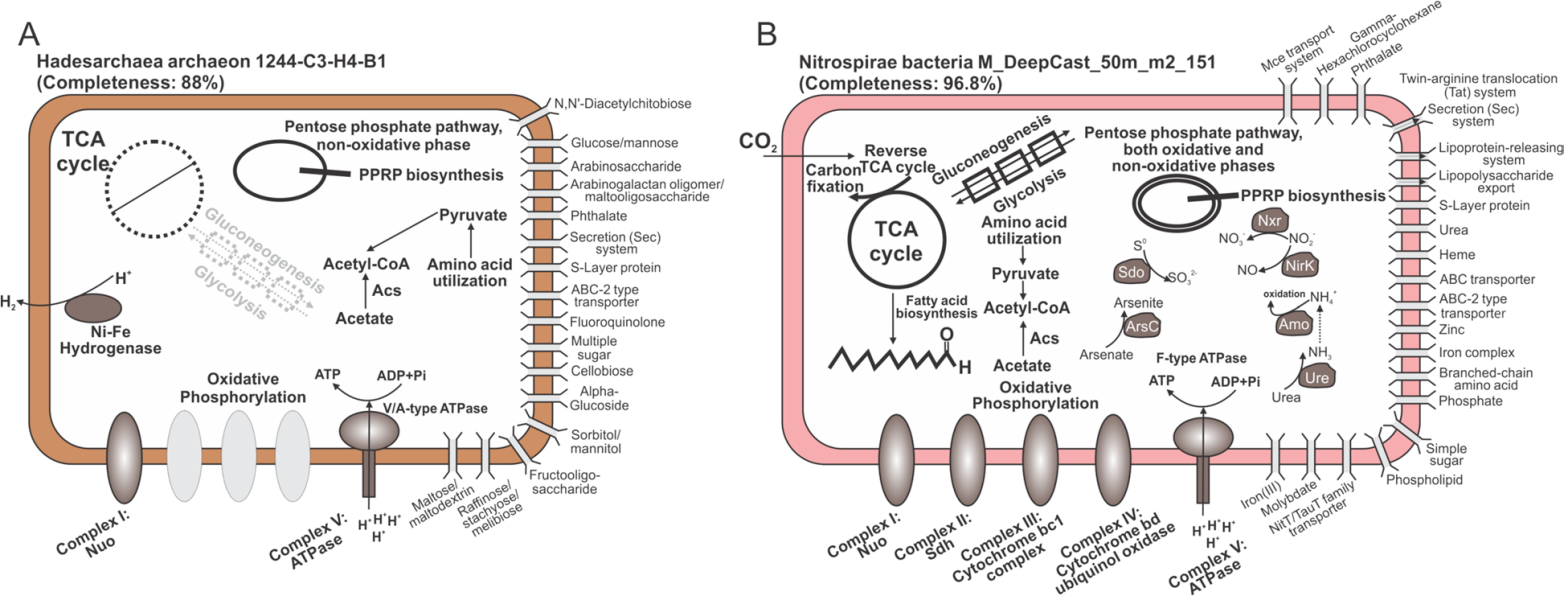
Cell metabolism diagrams of two microbial genomes. **A** Cell metabolism diagram of Hadesarchaea archaeon 1244-C3-H4-B1. **B** Cell metabolism diagram of Nitrospirae bacteria M_DeepCast_50m_m2_151. The absent functional pathways/complexes were labeled with dash lines

To demonstrate applications of reconstructing and depicting cell metabolism based on METABOLIC results, two microbial genomes were used as an example (Fig. 9). As illustrated in Fig. 9A, Hadesarchaea archaeon 1244-C3-H4-B1 has no TCA cycling gene components, which is consistent with previous findings in archaea within this class [79]. Gluconeogenesis/glycolysis pathways are also lacking in the genome; since gluconeogenesis is the central carbon metabolism responsible for generating sugar monomers which will be further biosynthesized to polysaccharides as important cell structural components [80], the lack of this pathway could be due to genome incompleteness. As an enigmatic archaeal class newly discovered in the recent decade, Hadesarchaea have distinctive metabolisms that separate them from conventional euryarchaeotal groups. They almost lost all TCA cycle gene components for the production of acetyl-CoA; while they could metabolize amino acids in a heterotrophic lifestyle [79]. It is posited that the Hadesarchaea genome has been subjected to a streamlining process possibly due to nutrient limitations in their surrounding environments [79]. Due to their metabolic novelty and limited available genomes at the current time, there are still uncertainties on unknown/hypothetical genes and pathways and unclassified metabolic potential across the whole class. The previous metabolic characterization on four Hadesarchaea genomes indicates that Hadesarchaea members could anaerobically oxidize CO, and H_2_ was produced as the side product [79]. In the Hadesarchaea archaeon 1244-C3-H4-B1 genome, METABOLIC results indicate the loss of all anaerobic carbon-monoxide dehydrogenase gene components, which suggests the distinctive metabolism of this Hadesarchaea archaeon from others and highlights the accuracy of METABOLIC in reflecting functional details.

We also reconstructed the metabolism for Nitrospirae bacteria M_DeepCast_50m_m2_151, a member of the Nitrospirae phylum reconstructed from Lake Tanganyika [56] (Fig. 9B). It contains the full pathway for the TCA cycle and gluconeogenesis/glycolysis. Furthermore, it also has the full set of oxidative phosphorylation complexes for energy conservation and functional genes for nitrite oxidation to nitrate. Other nitrogen cycling metabolisms identified in this genome include ammonium oxidation, urea utilization, and nitrite reduction to nitric oxide. The reverse TCA cycle pathway was identified for carbon fixation. The metabolic profiling result is in accord with the fact that Nitrospirae is a well-known nitrifying bacterial class capable of nitrite oxidation and living an autotrophic lifestyle [80]. Additionally, their more abundant distribution in nature compared to other nitrite-oxidizing bacteria such as *Nitrobacter* indicates their significant contribution to nitrogen cycling in the environment [80]. This highlights the ability of METABOLIC in reflecting functional details of more common and prevalent microorganisms compared to the Hadesarchaea archaeon. Notably as discovered from METABOLIC analyses, this bacterial genome also contains a wide range of transporter enzymes on the cell membrane, including mineral and organic ion transporters, sugar and lipid transporters, phosphate and amino acid transporters, heme and urea transporters, lipopolysaccharide and lipoprotein releasing system, bacterial secretion system, etc., which indicates its metabolic versatility and potential interactive activities with other organisms and the ambient environment. Collectively, METABOLIC result of functional profiling provides an intuitively-represented summary of a single microbial genome which enables depicting cell metabolism for better visualizing the functional capacity.

### METABOLIC accurately represents metabolism in the human microbiome

**Fig. 10:**
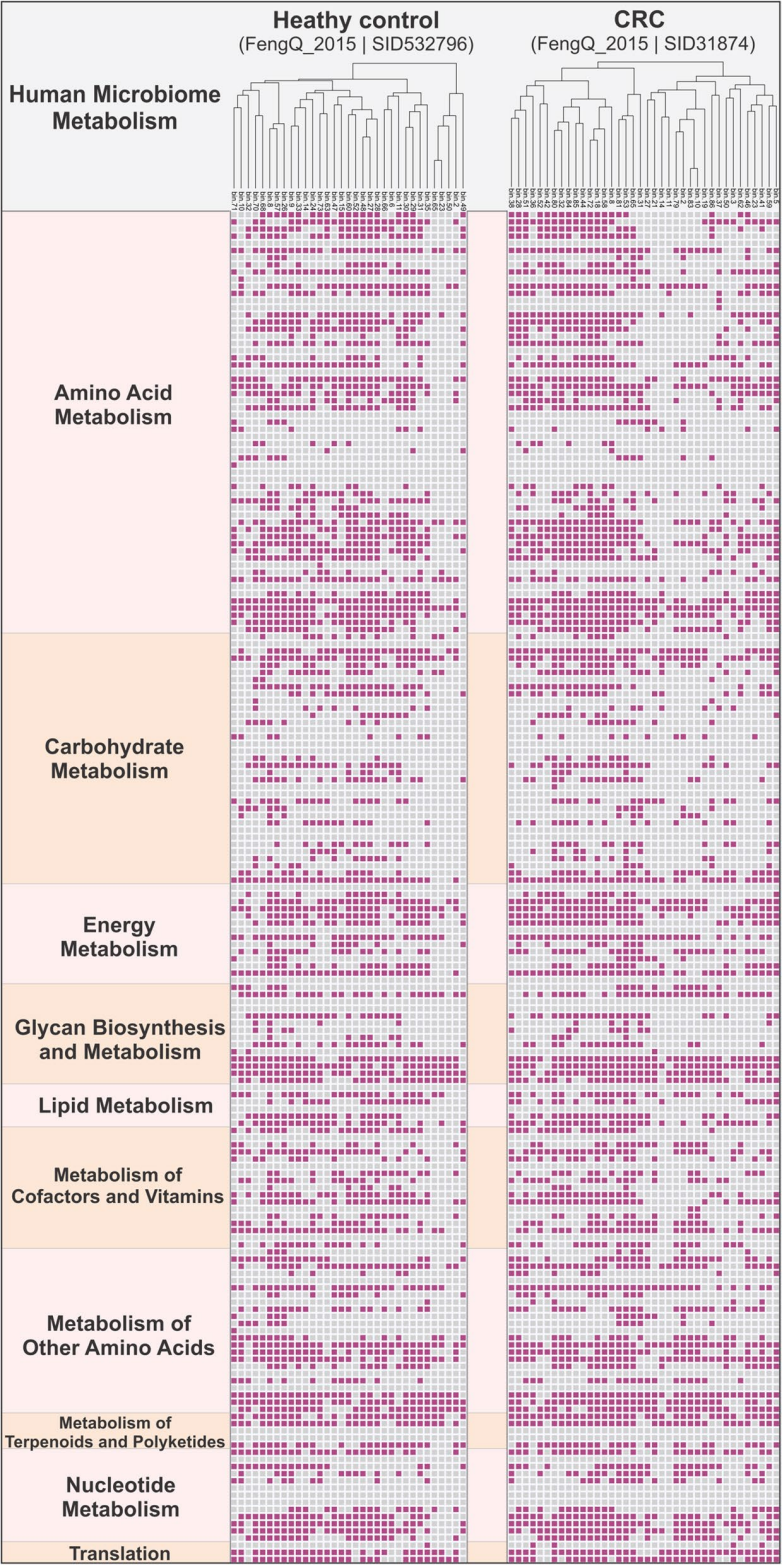
Presence/absence map of human microbiome metabolisms of a colorectal cancer (CRC) patient and a healthy control gut sample. The heatmap has summarized 189 horizontal entries (189 lines) based on 139 key functional gene families that covered 10 function categories. Purple cells indicate presence and gray cells indicate absence. Detailed KEGG KO identifier IDs and protein information for each function category were described in Supplementary Dataset S2

In addition to resolving microbial metabolism and biogeochemistry in environmental microbiomes, METABOLIC also accurately identifies metabolic traits associated with human microbiomes. The implications of microbial metabolism on human health largely remain a black box, much like microbial contributions to biogeochemical cycling. We demonstrate the utility of METABOLIC in human microbiomes using publicly available data from stool samples collected from patients with colorectal cancer and healthy individuals. From this study, we selected stool metagenomes from one colorectal cancer (CRC) and an age and sex-matched healthy control to conduct the comparison. The heatmap indicates the human microbiome functional profiles of both samples based on the marker gene presence/absence patterns (Fig. 10). As an example of METABOLIC’s application, we demonstrate that there were 28 markers with variations > 10% in terms of the marker-containing genome fractions between these two samples (Fig. 10, Additional file 13: Dataset S6). These 28 markers involved all the ten metabolic categories except for lipid metabolism and translation, suggesting the broad functional differences between these two samples. In addition to analyzing human microbiome-specific functional markers, METABOLIC can be used to visualize elemental nutrient cycling and analyze metabolic interactions in human microbiomes. Overall, it enables systematic characterization of the composition, structure, function, and interaction of microbial metabolisms in the human microbiome and facilitates omics-based studies of microbial community on human health [60].

## Discussion

The rapid increase in the availability of sequenced microbial genomes, metagenome-assembled genomes, and single-cell genomes has significantly benefited ecogenomic research on unraveling microbial functional roles and their metabolic contribution to biogeochemical cycles. Tools that enable to conduct accurate and reproducible functional profiling on genomic blueprints at the scale of both individual microorganisms and the whole microbial community offered significant applications and advances. They are fundamental to facilitate understanding of community-level functions, activities, interactions, and functional contributions in the era of multi-omics. An ideal tool for microbial biogeochemical profiling needs consideration on better organizing, interpreting, and visualizing the functional profile information; this is especially important for dealing with thousands of genomes reconstructed from metagenomes and studying community-scale interactive metabolisms. Meanwhile, fast, accurate, robust performance, and wide usage of the tool will allow for providing reliability and efficiency.

Here, we developed METABOLIC for profiling metabolisms, biogeochemical pathways, and community-scale functional networks. Instead of solely depending on widely adopted protein annotation databases, in METABOLIC two additional steps were added in order to accurately predict protein functions and reconstruct metabolic pathways. First, for TIGRfam/Pfam/Custom HMM profile databases, default NC/TC thresholds are often set too low to avoid noisy signals especially for annotating proteins from large sets of metagenomes wherein similar protein families often co-exist. This frequently leads to misannotations. To avoid this, we collected hmmsearch scores of previous annotation results and plotted these scores as a function of all annotations, and manually curated NC/TC by specifically picking the sharpest decreasing interval as the adjusted cutoff. Second, the motif validation step involves comparing potential hits to a set of manually curated highly conserved amino acid residues. This helps to distinguish two protein families with high sequence identity but different functions which are often difficult to separate by HMM profile-based annotations. These two steps help to filter out non-specific and cross-talking hits of important functional proteins for downstream bioinformatic analyses. After obtaining predicted metabolic pathways, many other software/online servers mostly provide raw annotation results with overwhelming yet unorganized details on characterizing protein functions. For microbial ecologists, it is fundamental to provide organized and intuitive results to facilitate understanding on the whole landscape of biogeochemical cycling capacities. In METABOLIC, such a function was developed to enable visualizing the presence/absence state of each step of biogeochemical cycles for individual genomes and the whole microbial community. Combined with gene abundance information calculated by metagenomic read mapping, we can identify the relative abundance for each step of biogeochemical cycles. Furthermore, METABOLIC can also visualize sequential reaction patterns for important organic and inorganic compound transformations. This visualization function of METABOLIC is practical for representing the “metabolic handoff” scenario of within-community interactions [2]. METABOLIC can be implemented in human microbiome with the same performance. Recently, METABOLIC was applied to stool metagenomic samples from 667 individuals who either were healthy or had adenomas or carcinomas of the colon, to profile organic/inorganic sulfate reduction and sulfide production [81]. This has considerably enlarged the utility of METABOLIC in community-scale investigation on human microbiomes for purposes of systematic microbiota-disease studies.

Previously, the community networks reflected by microbial genomes mostly focused on modeling reactions that are linked by metabolizing substrates and generating products [15, 19, 26]. On the contrary, METABOLIC was developed for a different purpose to study microbially mediated biogeochemical processes. In METABOLIC, the community-scale functional network provides an intuitive perspective on the metabolic connectivity among biogeochemical/metabolic steps and microbial contributions to these functions. MW-score, a metric that was built based on the same notion and methodology, offers quantitative measurement for these connected functions. Combined together, they represent which functions are more centralized (connected with others) and important (weighted with higher relative abundance) in the co-sharing functional networks and which groups of microbial players contribute to these functions. Additionally, metabolic Sankey diagrams can be drawn to further visualize microbial group contributions to different functions and biogeochemical cycles. As gene coverages generated by metagenomic read mapping can be replaced by transcript coverages generated by transcriptomic read mapping, we broaden the usage in reflecting active function connections and weights. In practical applications, functional networks and MW-scores can be made in a standardized, reproducible, and normalized manner, so parallel comparisons between communities (or samples) are applicable. The visualized network and Sankey diagram can also offer intuitive representations of functional connections and microbial contribution at both individual function and community-scale levels by using customized color schemes. There are other read-based metagenomic profiling tools, e.g., MetaPhlAn [28] and MEGAN [82], that can study the taxonomical and functional composition of microbiome at the community-scale level. Compared to read-based approaches which largely depend on the comprehensiveness of reference databases to capture microbial organisms, METABOLIC depends on the annotation of MAGs that is free from the limitation of reference databases on novel and rare organism characterization. METABOLIC specifically provides additional functionalities on annotation validation, result organization, and visualization which are meaningful to give reliable and easily accessible functional profiling results for microbial ecologists and biogeochemists to have a comprehensive understanding on the whole landscape of biogeochemical cycling capacities.

## Conclusions

Metabolic functional profile of microbial genomes at the scale of individual organisms and communities is essential to have a comprehensive understanding of ecosystem processes, and as a conduit for enabling functional trait-based modeling of biogeochemistry. We have developed METABOLIC as a metabolic functional profiler that goes above and beyond current frameworks of genome/protein annotation platforms in providing protein annotations and metabolic pathway analyses that are used for inferring the contribution of microorganisms, metabolism, interactions, activity, and biogeochemistry at the community-scale. METABOLIC facilitates standardization and integration of genome-informed metabolism into metabolic and biogeochemical models. We anticipate that METABOLIC will enable easier interpretation of microbial metabolism and biogeochemistry from metagenomes and genomes and enable microbiome research in diverse fields.

## Supplementary Information

**Additional file 1:** **Figure S1.** METABOLIC result table report.

**Additional file 2:** **Figure S2.** Functional network diagram based on the transcriptomic dataset from a hydrothermal vent sample.

**Additional file 3:** **Figure S3.** Functional network diagram based on the transcriptomic dataset from a deep-sea background sample.

**Additional file 4:** **Figure S4.** Microbial Sankey diagram based on the transcriptomic dataset from a hydrothermal vent sample.

**Additional file 5:** **Figure S5.** Microbial Sankey diagram based on the transcriptomic dataset from a deep-sea background sample.

**Additional file 6:** **Figure S6.** Metabolic profile diagram of a subsurface microbial community from Rifle, Colorado, USA.

**Additional file 7:** **Figure S7.** Comparison of sulfur metabolism at the community scale level.

**Additional file 8:** **Dataset S1.** Conserved motif sequences and motif pairs used in our analyses.

**Additional file 9:** **Dataset S2.** Summary table of Human Microbiome Marker genes.

**Additional file 10:** **Dataset S3.** METABOLIC result of metagenomes from eight different environments.

**Additional file 11: Dataset S4.** Comparison of protein prediction performance among five software packages/online servers on the genome of *Escherichia coli* O157H7 str. Sakai.

**Additional file 12: Dataset S5.** Comparison of protein prediction performance among five software packages/online servers on the genome of *Pseudomonas aeruginosa* PAO1.

**Additional file 13:** **Dataset S6.** Human microbiome functional profiling results for a CRC subject and a healthy control.
