# Supplementary material for: METABOLIC: high-throughput profiling of microbial genomes for functional traits, metabolism, biogeochemistry, and community-scale functional networks
Source: Microbiome. 2022 Feb 16;10:33. doi: 10.1186/s40168-021-01213-8 (PMC8851854; doi:10.1186/s40168-021-01213-8)
Supplement: Supplementary file 4 — Additional file 3: Figure S3. Functional network diagram based on the transcriptomic dataset from a deep-sea background sample. [file 40168_2021_1213_MOESM4_ESM.pdf]

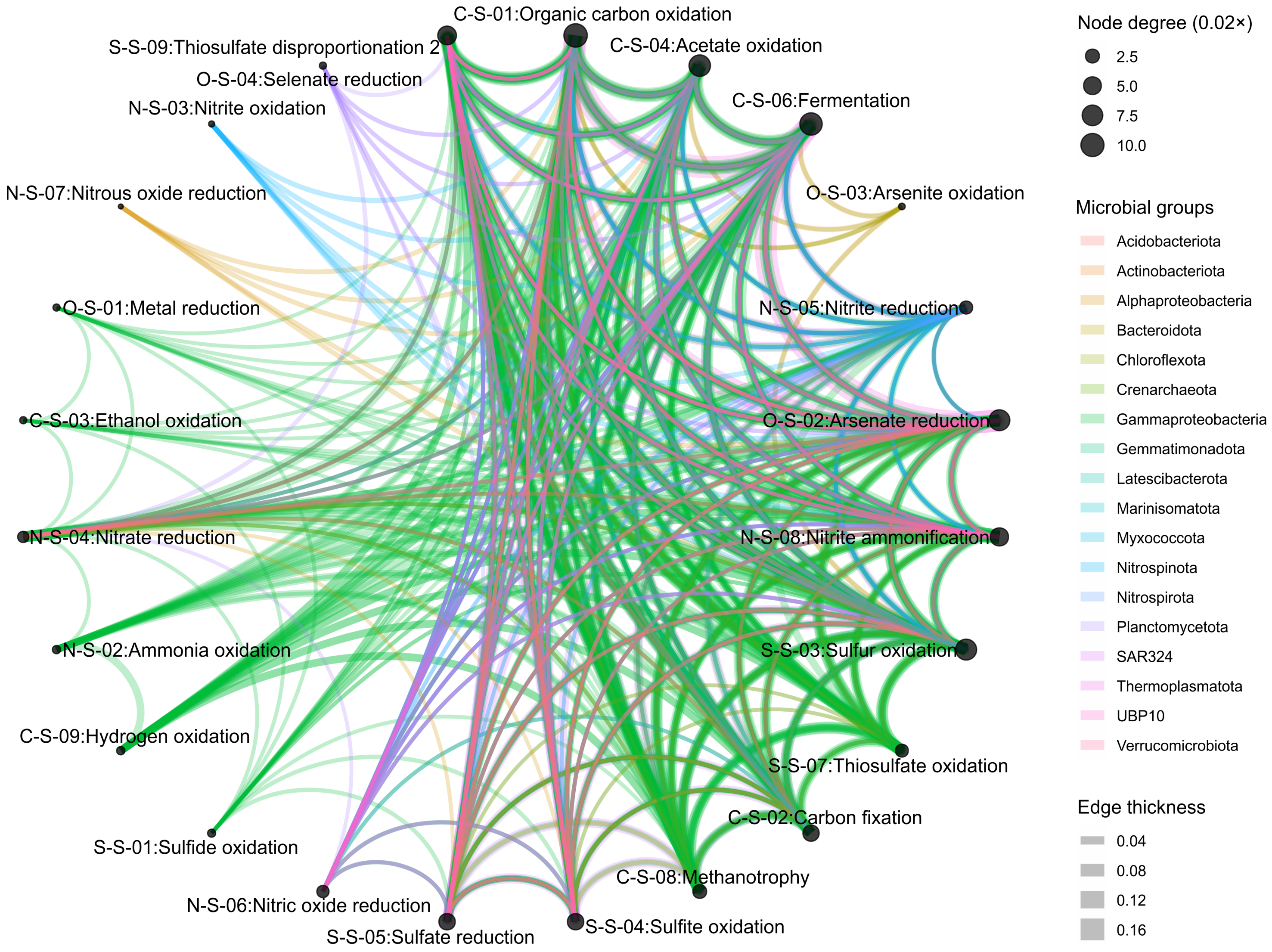

Supplementary Figure S3. Functional network diagram based on the transcriptomic dataset from a deep-sea background sample. The nodes represent biogeochemical cycling steps, and edges connecting two given nodes represent the functional connections between them. The size of the node was depicted according to the degree (number of connections to each node). The thickness of the edge was depicted according to the average of the gene expression values of the connected two biogeochemical cycling steps, which were calculated by the transcriptomic dataset from the Guaymas Basin hydrothermal background sample. The color of the edge was assigned based on the taxonomic group of the represented genome.
