# Supplementary material for: METABOLIC: high-throughput profiling of microbial genomes for functional traits, metabolism, biogeochemistry, and community-scale functional networks
Source: Microbiome. 2022 Feb 16;10:33. doi: 10.1186/s40168-021-01213-8 (PMC8851854; doi:10.1186/s40168-021-01213-8)
Supplement: Supplementary file 5 — Additional file 4: Figure S4. Microbial Sankey diagram based on the transcriptomic dataset from a hydrothermal vent sample. [file 40168_2021_1213_MOESM5_ESM.pdf]

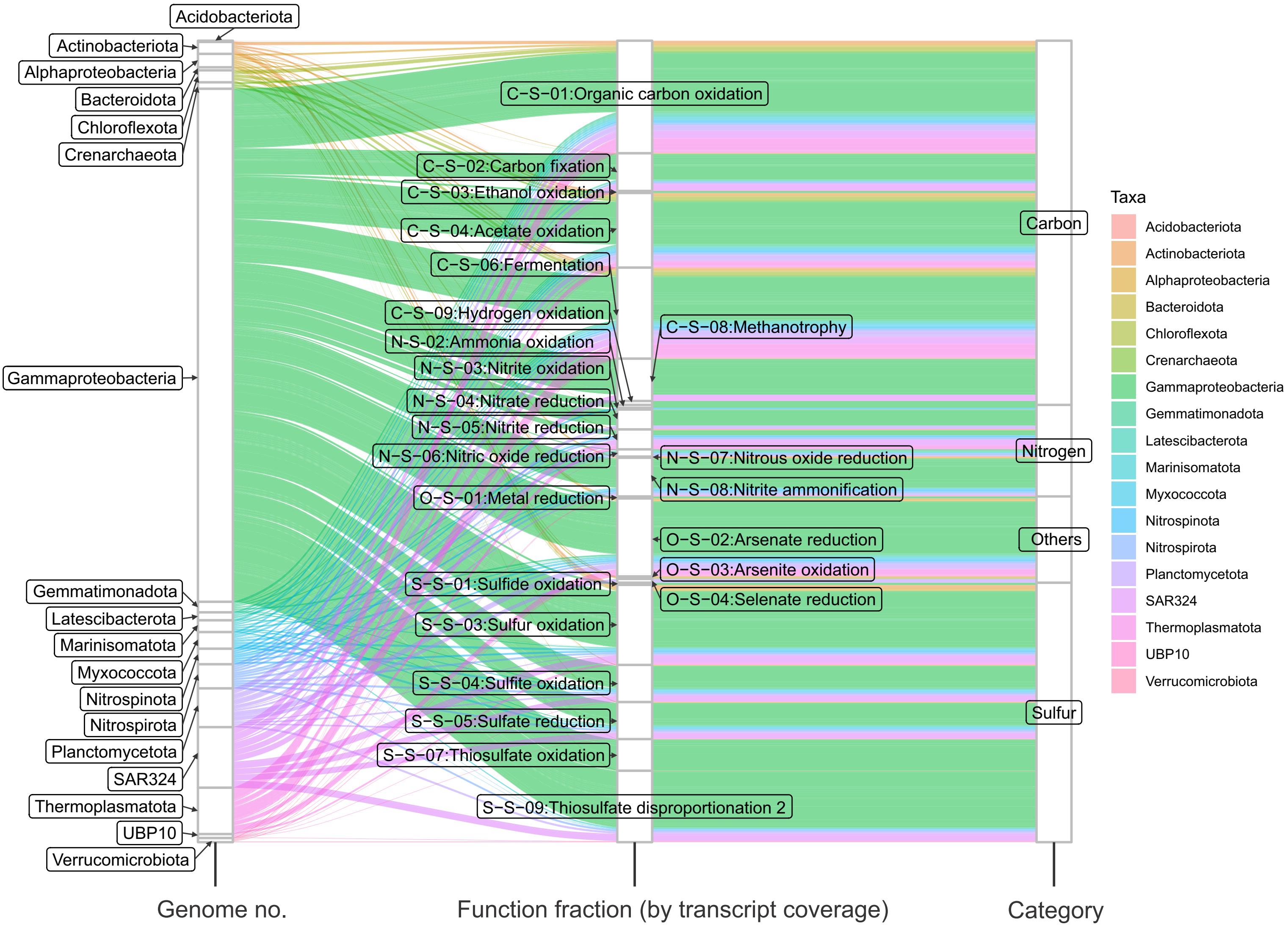

Supplementary Figure S4. Microbial Sankey diagram based on the transcriptomic dataset from a hydrothermal vent sample. The three columns of the Sankey diagram represent taxonomic groups and genome numbers, the expression fraction of each microbial group calculated by transcriptome coverage, and the function category.
