# Supplementary material for: METABOLIC: high-throughput profiling of microbial genomes for functional traits, metabolism, biogeochemistry, and community-scale functional networks
Source: Microbiome. 2022 Feb 16;10:33. doi: 10.1186/s40168-021-01213-8 (PMC8851854; doi:10.1186/s40168-021-01213-8)
Supplement: Supplementary file 7 — Additional file 6: Figure S6. Metabolic profile diagram of a subsurface microbial community from Rifle, Colorado, USA. [file 40168_2021_1213_MOESM7_ESM.pdf]

Category	Genome Group ID	G01	G02	G03	G04	G05	G06	G07	G08	G09	G10	G11	G12	G13	G14	G15	G16	G17	G18	G19	G20	G21	G22	G23	G24	G25	G26	G27	G28	G29	G30	G31	G32	G33	G34	G35	G36	G37	G38	G39	G40
	**Genome Number**	12	3	16	3	36	7	1	6	6	4	8	4	1	19	1	1	5	5	61	1	1	1	3	7	2	2	3	8	1	9	6	7	2	1	9	2	5	1	11	2
	Thermophilic specific																																			1					
C metabolism	Amino acid utilization	11	3	15	2	36	4	1	6	5	3	8	4	1	19	1	1	5	5	61	1	1	1	3	6	2	2	3	8		9		7	2	1	9	2	4	1	10	1
C metabolism	Aromatics degradation	9	1	8	2	20	1		6	2	3	7	4		4			1	5	46	1		1	2		2	1	3	6		4		2	2	1	1	1	3	1	2	1
C metabolism	Complex carbon degradation	7	1	10	2	29	3	1		1		3	1		4	1	1	3	3	27	1	1	1	3		2			2	1		1	4			3			1	10	1
C metabolism	Fermentation	11	3	7	3	35	7	1	6	6	2	8	4	1	19	1	1	5	5	44	1	1	1	3	7	2	2	3	8	1	7	3	6	2	1	9	2	1	1	10	2
C metabolism	C_1 metabolism|Aerobic CO oxidation	9	1	3	1	8	2	1		4					9			1		23			1			1		1	1		1				1	1			1	1	1
C metabolism	C_1 metabolism|Formaldehyde oxidation			10																32																					
C metabolism	C_1 metabolism|Formate oxidation	7	1	4	1	21	3	1		4		7	4		1					32		1	1	3	4	2	1	1	6		2		2		1	5	2		1	4	2
C metabolism	C_1 metabolism|Methanol oxidation	4		8					1											14																					
C metabolism	C_1 metabolism|Methyl amine -> formaldehyde			3																1																					
C metabolism	Methane oxidation			4		2									3				1	12					1		1				2	1	1							1	1
C metabolism	Methane production																																								
C metabolism	Carbon fixation|3 Hydroxypropionate cycle	1		4		3						3								22						1			2							2					
C metabolism	Carbon fixation|3HP/4HB	2	3	2	1	2				1		3								1			1	1		2					1					2					1
C metabolism	Carbon fixation|CBB cycle - Rubisco			10																29																					
C metabolism	Carbon fixation|Reverse TCA cycle						1		6						7													2	3			1									
C metabolism	Carbon fixation|Wood-Ljungdahl pathway	1	2		1	3	1			5	2	7	3		8	1	1	1	3			1		2	2				5		2		5			6	1			7	
N metabolism	Nitrogen cycling|Ammonia oxidation																																								
N metabolism	Nitrogen cycling|Anammox																																3								
N metabolism	Nitrogen cycling|N_2 fixation								4		1	5	3		2					8					1		1		5					2		4			1	2	
N metabolism	Nitrogen cycling|Nitrate reduction	5		6	1	7	1		5	2	1	4	2		2				3	33	1			2		2	1		2		1			2	1		2				
N metabolism	Nitrogen cycling|Nitric oxide reduction	8		4	1	15	5		5			3	1		1				1	23	1		1	3		2	1	1	4		5		2		1	2			1		2
N metabolism	Nitrogen cycling|Nitrite oxidation	6			1	2				1					3				1	3			1			2	1	1		1	6		1		1		1	3	1	1	1
N metabolism	Nitrogen cycling|Nitrite reduction	4	1	4		4	4		3	3		1	2						3	25						1		2	3	1	1	1	1				1	3			1
N metabolism	Nitrogen cycling|Nitrite reduction to ammonia	3		8	2	21	4			2		6	4		8				2	39	1	1	1	3		1	2	1	8		4		2		1	3	1	4		3	1
N metabolism	Nitrogen cycling|Nitrous oxide reduction	2			2	9			3	1					3					1					1						1										
S metabolism	Sulfur cycling|Sulfate reduction	8	1	12	2	24	1		5	2	3	8	2		5		1		5	49			1	3	1	2	2	2	6		7	1	3	2	1	4		4	1	8	
S metabolism	Sulfur cycling|Sulfide oxidation		1	5		4	1		6			1			1				3	23									3												
S metabolism	Sulfur cycling|Sulfite reduction (Asr)																																								
S metabolism	Sulfur cycling|Sulfite reduction (Dsr)																																								
S metabolism	Sulfur cycling|Sulfur oxidation	6		13	3	13	1	1		3	4	8	4	1	13		1	1	4	41	1	1		3	2	2	2	2	6	1	7		7	2	1	2	1	4	1	2	2
S metabolism	Sulfur cycling|Sulfur reduction																																								
S metabolism	Sulfur cycling|Thiosulfate disproportionation																																								
S metabolism	Sulfur cycling|Thiosulfate oxidation	2		1					6											26																					
Other metabolisms	Hydrogenases	4	3	5	1	8	2		6	5	2	7	3		11	1	1	1	3	22		1	1	3	2	2		2	8		3	1	5			6	1			7	1
Other metabolisms	Urea utilization	2		5									1							24						1	1		2											1	
Other metabolisms	Halogenated compound utilization	4	1	3		4				2		1								25			1			2		1								1	1	1	1	6	
Other metabolisms	Perchlorate reduction	2		2		5	1		3	2	1		1		1				3	7	1			2				2	1		1							1			
Other metabolisms	Chlorite reduction	3		1	1	3			5			4	1							8						2	1		2					2							
Other metabolisms	As cycling|Arsenate reduction	10	1	12	3	34	7	1	5	6	2	7	3	1	18	1	1	3	2	45	1	1	1	3	6	2	2	1	6		5		4	2	1	8		3	1	9	1
Other metabolisms	As cycling|Arsenite oxidation			2	1								1						1	5						1															
Other metabolisms	Selenate reduction	1	1		2	13	2	1		4		2			10			1		2							1						2		1	3				4	
Other metabolisms	Nitrile hydration			1																6			1			1															
Other metabolisms	Metal reduction	10			2	1	2					3	3		3				2	21			1	3		2	2	1	2						1			1	1		2

Genome group map:

G01	Acidobacteriota	G11	Desulfobacterota	G21	KSB1	G31	Patescibacteria
G02	Actinobacteriota	G12	Desulfuromonadota	G22	Latescibacterota	G32	Planctomycetota
G03	Alphaproteobacteria	G13	Edwardsbacteria	G23	MBNT15	G33	SAR324
G04	Bacteria|UKC	G14	Elusimicrobiota	G24	Margulisbacteria	G34	Schekmanbacteria
G05	Bacteroidota	G15	Fibrobacterota	G25	Methylomirabilota	G35	Spirochaetota
G06	Bdellovibrionota	G16	Firestonebacteria	G26	Myxococcota	G36	Thermoplasmata
G07	Caldatribacteriota	G17	Firmicutes	G27	Nitrospinota	G37	UBA10199
G08	Campylobacterota	G18	GWC2-55-46	G28	Nitrospirota	G38	UBP10
G09	Chloroflexota	G19	Gammaproteobacteria	G29	O2-12-FULL-43-9	G39	Verrucomicrobiota
G10	Cyanobacteriota	G20	Gemmatimonadota	G30	Omnitrophota	G40	Zixibacteria

Supplementary Figure S6. Metabolic profile diagram of a subsurface microbial community from Rifle, Colorado, USA. The dereplicated genomes from the Rifle subsurface were assigned with genome taxonomy by GTDB-Tk, and were clustered into 40 microbial groups. The metabolic functional traits were summarized and represented accordingly.
