# Supplementary material for: METABOLIC: high-throughput profiling of microbial genomes for functional traits, metabolism, biogeochemistry, and community-scale functional networks
Source: Microbiome. 2022 Feb 16;10:33. doi: 10.1186/s40168-021-01213-8 (PMC8851854; doi:10.1186/s40168-021-01213-8)
Supplement: Supplementary file 8 — Additional file 7: Figure S7. Comparison of sulfur metabolism at the community scale level. [file 40168_2021_1213_MOESM8_ESM.pdf]

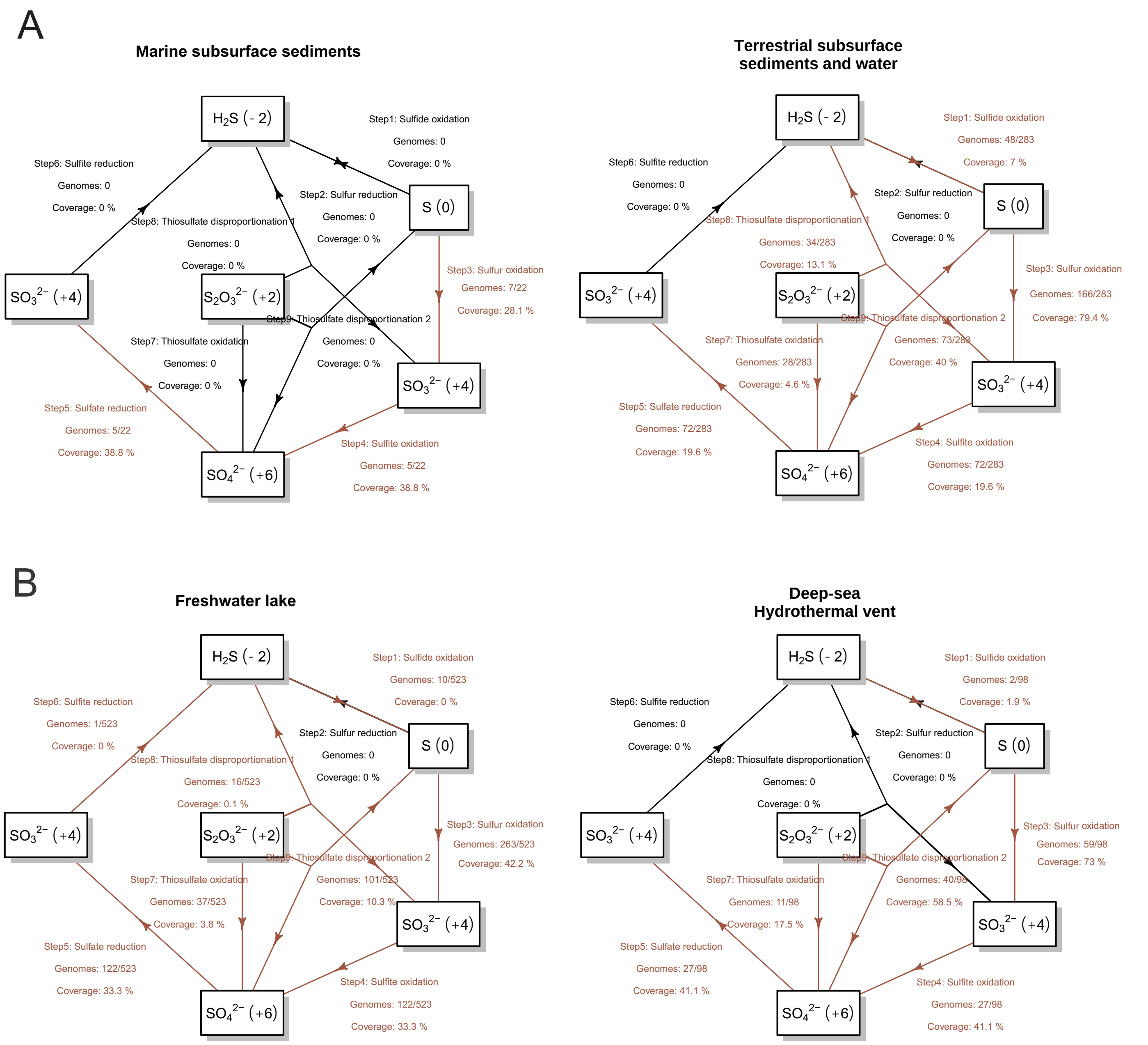

Supplementary Figure S7. Comparison of sulfur metabolism at the community scale level. (A) Comparison between deep subsurface sediments (marine subsurface) and Rifle (terrestrial subsurface). (B) Comparison between Lake Tanganyika (freshwater) and Guaymas Basin (deep-sea plume). Each arrow represents a single transformation/step within a cycle. Indicated above the arrows in sequential order from top to bottom are: Step number and reaction, number of genomes that can conduct these reactions (and the total genome number of the community), metagenomic coverage expressed as a percentage of the community. The dark red labeled arrows and tags indicate the presence of the transformation/step within the community.
