# Supplementary material for: METABOLIC: high-throughput profiling of microbial genomes for functional traits, metabolism, biogeochemistry, and community-scale functional networks
Source: Microbiome. 2022 Feb 16;10:33. doi: 10.1186/s40168-021-01213-8 (PMC8851854; doi:10.1186/s40168-021-01213-8)
Supplement: Supplementary file 2 — Additional file 1: Figure S1. METABOLIC result table report. [file 40168_2021_1213_MOESM2_ESM.pdf]

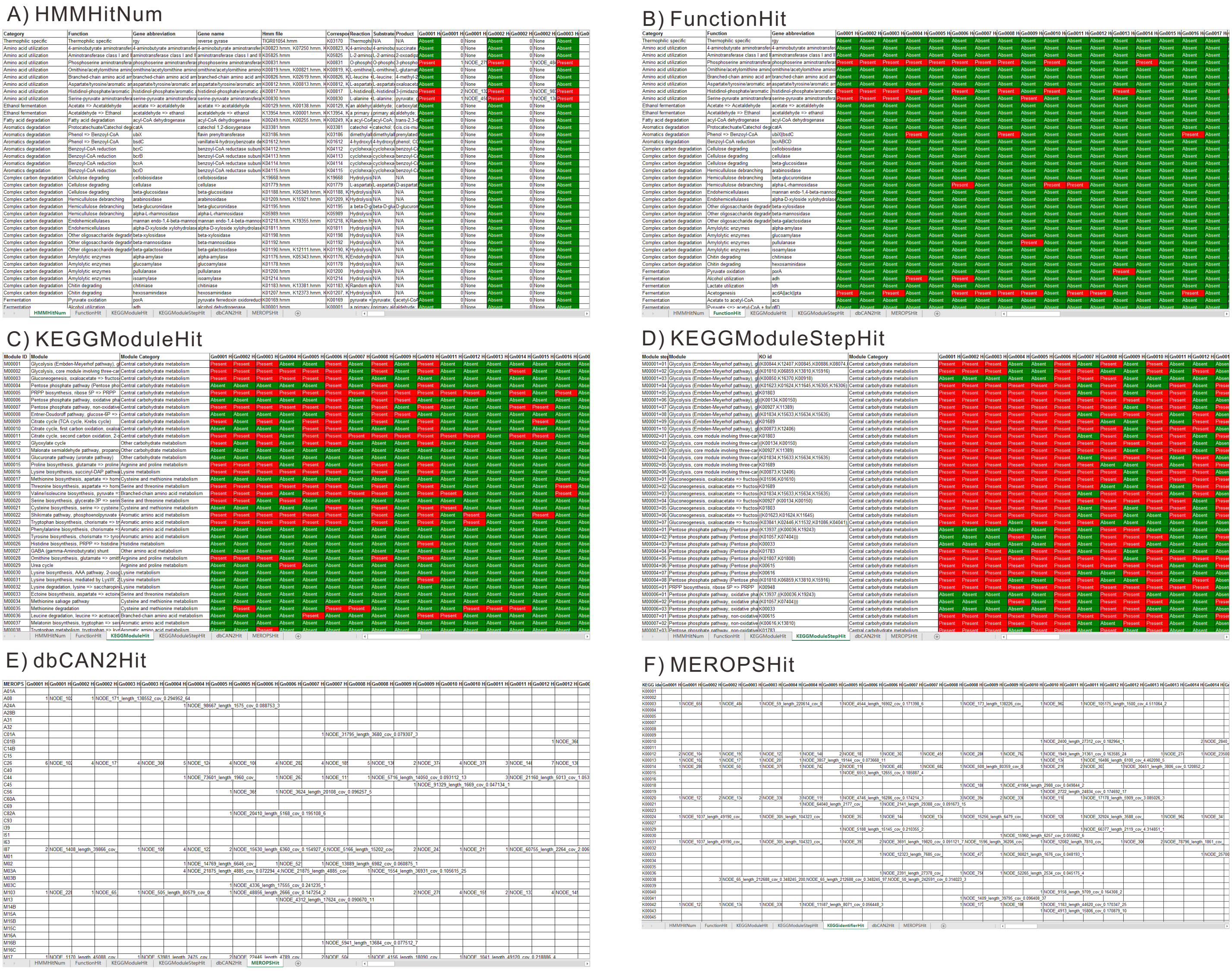

Supplementary Figure S1. METABOLIC result table report. A) “HMMHitNum” worksheet represents detailed functional information and reflects the presence/absence of an HMM profile, HMM hit protein numbers, and IDs. B) “FunctionHit” worksheet reflects the presence/absence of a functional trait. C) “KEGGModuleHit” worksheet reflects the presence/absence of a KEGG module by given a module cutoff value. D) “KEGGModuleStepHit” worksheet reflects the presence/absence of a KEGG module step. E) “dbCAN2Hit” worksheet reflects the hit numbers and names of CAZymes. F) “MEROPSHit” worksheet reflects the hit numbers and names of peptidases and inhibitors.
